# Growth of fatty acid vesicles coupled with amino acid sequences of peptides toward evolvable protocells

**DOI:** 10.1038/s42004-026-02043-1

**Published:** 2026-04-30

**Authors:** Akiko Baba, Kazuki Yokoyama, Keidai Sato, Shuna Asanuma, Tomoko Kawahata, Ulf Olsson, Daisuke Unabara, Tasuku Hamaguchi, Koji Yonekura, Masayuki Imai

**Affiliations:** 1 Department of Physics, Graduate School of Science, Tohoku University, Sendai, Japan; 2 Division of Physical Chemistry, Department of Chemistry, Lund University, Lund, Sweden; 3 Institute of Multidisciplinary Research for Advanced Materials, Tohoku University, Sendai, Japan; 4 Biostructural Mechanism Laboratory, RIKEN SPring-8 Center, Hyogo, Japan

**Keywords:** Origin of life, Membranes, Biophysical chemistry, Self-assembly, Self-assembly

## Abstract

Understanding how genetic polymer sequences became coupled with the reproduction of protocellular compartments is a fundamental challenge in the study of the emergence of living systems. Here, we demonstrate that the coexistence of peptides with defined amino acid sequences and fatty acid vesicles can establish a primitive form of this coupling. We prepared systematically sequence-controlled peptides and examined how their sequences influence the growth rate (fitness) of fatty acid vesicles. The growth of fatty acid vesicles was estimated by dynamic light scattering (DLS) and cryogenic transmission electron microscopic techniques when fatty acid molecules and peptides were fed into a fatty acid vesicle suspension. The relationship between amino acid sequences of peptides and vesicle growth rate was visualized as a fitness landscape, which reveals that specific amino acid sequences promote vesicle growth significantly. Furthermore, we observed epistasis, where the effect of amino acid residue replacement on the fitness depends on the remaining amino acid sequence. Finally, we show that vesicle growth is thermodynamically driven by peptide-induced modulation of the chemical potential of fatty acid molecules. These findings provide direct experimental evidence that primitive sequence information can become spontaneously coupled to vesicle growth.

## Abstract (web summary)

Understanding how genetic polymer sequences couple to protocellular reproduction is central to unraveling life’s origins. Here, the authors show that specific peptide sequences promote the growth of fatty acid vesicles, revealing a primitive coupling mechanism and providing insight into the chemical processes that may have driven early protocell evolution.

## Introduction

Living organisms proliferate themselves by reproducing both the membrane-based containers and the genetic polymers within. The heart of proliferation lies in the central dogma in which the nucleotide sequence in nucleic acids is translated into the amino acid sequence in proteins, and the amino acid sequence determines the ability to catalyze metabolic reactions to synthesize membrane molecules^1^. If a mutation of nucleic acids results in an amino acid sequence of the protein with higher catalytic ability, the probability of reproductive success (fitness) increases, leading to Darwinian evolution. Therefore, a fundamental challenge concerning the emergence of living organisms is to elucidate how the sequence of the genetic polymer is coupled with the reproduction of the membrane-based containers, *i.e*., vesicles^2^.

One plausible scenario for the emergence of this coupling is that protometabolic reaction networks were generated from primitive genetic polymers. The protometabolic reaction networks eventually synthesized membrane molecules in a manner dependent on the sequence of those genetic polymers. The membrane molecules then self-assembled to form vesicles, which underwent growth and division (reproduction)^3–5^. However, even for the synthesis of simple membrane molecules, the metabolic pathway would be highly complex requiring the support of many enzymes. Therefore, the emergence of such a pathway to synthesize membrane molecules through random processes is highly improbable, and the driving force behind this process remains a great mystery.

On the other hand, prebiotic molecules, such as fatty acids, peptides, and nucleosides, were presumably supplied to the early Earth from various sources (delivery by meteorites, synthesis on mineral surfaces, and electrochemical synthesis by spark discharge)^6^ and dispersed in the primordial soup^7^. When the concentration of fatty acids in the solution exceeds the critical vesicular concentration (CVC), they begin to form vesicles. The coexisting primitive peptides and nucleosides might have promoted vesicle growth by incorporating fatty acids in the external solution and eventually reproduction in a manner dependent on the sequence of the primitive polymers^8–18^. Thus, the coupling of the sequence of genetic polymers with vesicle reproduction could have emerged prior to the evolution of protometabolic pathways capable of synthesizing fatty acids. In this study, we set out to explore this scenario by demonstrating that the coexistence of fatty acid vesicles and peptides leads to vesicle growth depending on the amino acid sequence of peptides.

The relationship between the amino acid sequences of peptides and the growth rate of fatty acid vesicles is analyzed based on the concept of the fitness landscape^19–23^. In prebiotic protocell models, fitness may be assessed by vesicle reproduction rate, which includes both vesicle growth and division. While vesicle division can be induced by incorporating two types of membrane molecules with different molecular shapes^17,18,24–26^, introducing additional types of fatty acids into the present system would increase complexity and potentially reduce stability. Therefore, in this study, the growth rate of vesicles is used as a proxy for fitness. To evaluate whether fatty acid vesicle–peptide protocells can support the emergence of evolvable systems, we characterize how peptide sequence controls vesicle fitness from three essential perspectives. First, we show that dipeptides with specific amino acid sequences strongly promote vesicle growth, analogous to sequence motifs in modern proteins. Second, we find that the growth-promoting effect of these motifs is largely preserved when the motif is embedded in longer peptides, from di- to tri- and tetrapeptides. Third, systematic amino acid substitutions reveal strong sequence-dependent changes in fitness, demonstrating epistasis in the sequence–growth relationship. The epistasis contributes to sequence-dependent variation in protocell fitness, generating diversity among vesicle−peptide systems^22,23,27,28^. Although our experimental system does not include peptide synthesis pathways, we ask whether fatty acid vesicle−peptide protocells already exhibit basic sequence–fitness properties that are characteristic of evolvable systems.

To investigate how peptide sequences influence fatty acid vesicle growth, we use sequence-defined peptides as model genetic polymers and decanoic acid/decanoate (DA) vesicles as model primitive vesicles. DA was chosen because it forms stable vesicles under prebiotic conditions and its physicochemical properties are well characterized^29–32^. We first quantify DA vesicle growth upon addition of DA micelles using dynamic light scattering (DLS) and cryogenic transmission electron microscopy (cryo-TEM). We then examine how single amino acids affect vesicle growth and identify physicochemical factors underlying growth promotion. Next, we analyze the sequence−fitness relationship using di-, tri-, and tetrapeptides, showing that specific sequences strongly enhance vesicle growth (property 1), that amino-acid substitutions have sequence-dependent effects (property 3; epistasis), and that these sequence–fitness relationships are largely retained when motifs are embedded in longer peptides (property 2), supported by Walsh analysis^33–36^. Finally, we relate these sequence effects to peptide−DA interactions that provide the physical driving force for vesicle growth and discuss their implications for the emergence of living systems.

## Results and discussion

### Growth of DA vesicles by addition of DA micellar solution

**Fig. 1:**
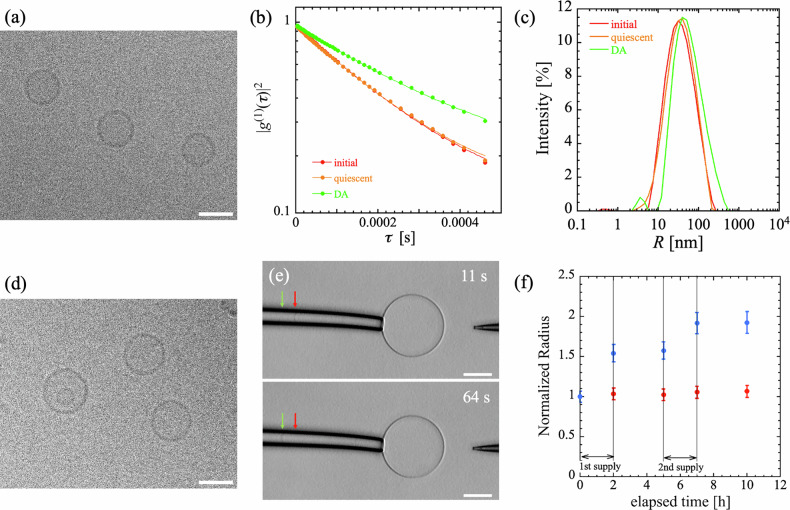
Growth of DA SUVs by addition of DA micellar solution. **a** Cryo-TEM micrograph of DA vesicles just before the vesicle growth (initial: *t* = 0 h). Scale bar indicates 30 nm. **b** Intermediate scattering functions (at *q* = 2.22 $\times$ 10^7^ m^-1^, *q*: absolute value of scattering vector) for DA SUVs at initial state (*t* = 0 h: red), after 2 hours in the quiescent state (*t* = 2 h: orange), and immediately after the addition of 100 mM DA micellar solution (*t* = 2 h: green). Solid lines indicate results of fitting using Eq. (1). **c** Size distribution functions obtained by inverse Laplace transformation of intermediate scattering functions using a constrained regularization program, CONTIN, for DA SUVs at initial state (*t* = 0 h: red solid line), after 2 h in the quiescent state (*t* = 2 h: orange solid line), and immediately after the addition of 100 mM DA micellar solution (*t* = 2 h: green solid line). A small additional peak at apparent size below ~5 nm observed in the sample supplying DA micelles is not considered to represent vesicles but is attributed to small DA aggregates and/or noise in the DLS analysis. **d** Cryo-TEM micrograph of DA vesicles just after the vesicle growth with supplying DA micelles (*t* = 2 h). Scale bar indicates 30 nm. **e** Increase in vesicle membrane area upon injection of DA micelles. A DA vesicle was held with a holding pipette, and a 100 mM DA micellar solution was injected from a distance of 20 μm. The upper image shows the vesicle 11 s after the start of injection, with the end of the vesicle inside the pipette indicated by a red arrow. The lower image shows the vesicle after 64 s, with the new position of the vesicle end indicated by a green arrow. Scale bar indicates 20 μm. The full-time course of this growth process is shown in the Supplementary Movie. **f** Time evolution of normalized vesicle radius, $\bar{R}/{\bar{R}}_{{{\rm{ini}}}}$ (${\bar{R}}_{{{\rm{ini}}}}$; mean vesicle radius at initial state) of DA SUVs in the quiescent state (red circles) and after addition of 100 mM DA micellar solution (blue circles). DA micellar solution was supplied from *t* = 0 to 2 h (1st supply) and from 5 to 7 h (2nd supply). The error bars indicate standard error of the mean (SEM) estimated from three measurements at different scattering angles.

First, we examine the growth of DA vesicles by supplying DA micelles without amino acids or peptides as a reference state. We prepared 100 mM DA small unilamellar vesicles (SUVs) suspensions with pH 7.7, where the CVC is around 60 mM. The obtained DA SUVs were characterized by cryo-TEM observation. As shown in Fig. 1a, most of initial SUVs had a spherical and unilamellar structure with the thickness of ~1.5 nm, although they had large size polydispersity. Therefore, the mean vesicle size and the size distribution were estimated by DLS^37^ technique, since the statistical average of SUVs within the scattering volume (approximately 1 mm³) is obtained. The intermediate scattering function, ${g}^{\left(1\right)}(\tau )$, for initial SUVs (*t* = 0 h) is shown by red circles in Fig. 1b and expressed by

1 $${g}^{\left(1\right)}\left(\tau \right)={\int }_{0}^{\infty }\exp \left(-\varGamma \tau \right)G\left(\varGamma \right)d\varGamma \cong \exp \left(-\bar{\varGamma }\tau \right)$$

where *Γ* is the initial relaxation rate, $G(\varGamma )$ is the distribution function of *Γ*, and $\bar{\varGamma }$ is the mean initial relaxation rate. Here, we assume that the initial relaxation rate is governed by the diffusion of the center of mass of spherical SUVs. Then, the mean diffusion constant, $\bar{D}$, is expressed by $\bar{\varGamma }=\bar{D}{q}^{2}$, where *q* is the absolute value of the scattering vector. Using the Stokes–Einstein equation for spherical vesicles, $\bar{D}={k}_{{{\rm{B}}}}T/5\pi \eta \bar{R}$ (*k*_B_; Boltzmann constant, *T*; temperature, and *η*; viscosity of medium), we obtain the mean hydrodynamic radius of SUVs, $\bar{R}$. The size distribution of the vesicles is obtained by inverse Laplace transform (CONTIN analysis^38^) of ${g}^{\left(1\right)}\left(\tau \right)$.

The initial SUVs had the mean hydrodynamic radius, $\bar{R}$ = 49 nm and a broad size distribution ranging from 10 to 200 nm, as shown by the red peak profile in Fig. 1c (*t* = 0 h). The vesicle suspension was left quiescent to examine how the size and distribution of the vesicles changed. The intermediate scattering function (orange circles in Fig. 1b) and the size distribution function (orange profile in Fig. 1c) after 2 h under quiescent condition remained almost unchanged, indicating that no spontaneous vesicle growth occurred. Then, we supplied 100 mM DA micellar solution with pH 10 to the initial DA vesicle suspension with pH 7.7. It should be noted that since the critical micellar concentration (CMC) of the DA micellar solution at pH 10 is around 90 mM^31^, the DA molecules in 100 mM DA micellar solution are mostly dispersed as a single molecule state (unimer). The DA micellar solution was added slowly over 2 h with stirring, and 0.1 mL of 100 mM HCl solution was also added simultaneously to avoid pH change of the vesicle suspension, where the pH of the DA vesicle suspension after the feeding of the DA micellar solution was nearly constant at pH 7.7. As the pH of the added micellar solution decreased, the CMC decreased accordingly, and decanoate that could no longer stay dispersed in the aqueous phase became incorporated into the DA vesicle membrane, resulting in vesicle growth^39^. Changes in the vesicle shape by addition of DA micellar solution were examined by the cryo-TEM observation. After feeding the DA micellar solution, the DA vesicles retained their spherical and unilamellar structure although they have a large size polydispersity (Fig. 1d). The intermediate scattering functions of DA vesicles that were fed with 100 mM DA molecules are shown by green circles in Fig. 1b. After the feeding period, the relaxation rate decreased significantly, and the mean hydrodynamic radius of the DA vesicles increased from 49 nm to 70 nm during this period. The size distribution shifted toward larger sizes while maintaining its overall shape (green profile in Fig. 1c), suggesting vesicle growth due to DA incorporation.

To assess the validity of evaluating vesicle growth rates from changes in vesicle size before and after DA micelle supply, we performed the following two experiments. 1) Direct time-resolved observation of DA vesicle growth under constant feeding. DA giant vesicles were held on a holding pipette under a constant suction pressure, and a 100 mM DA micellar solution was injected at a constant rate. The observed vesicle growth is shown in Supplementary Movie. The increase in membrane area was visualized by the change in length of the cylindrical vesicle segment inside the pipette (Fig. 1e). The vesicle membrane area increased approximately linearly with time as shown in Supplementary Fig. 1. 2) Two-step feeding experiment. We supplied a 100 mM micellar solution to DA SUVs for 2 h, stopped the injection for 3 h, and then resumed feeding for another 2 h. DLS measurements showed that the normalized vesicle radius increased from ${\bar{R}}_{t=0}/{\bar{R}}_{t=0}=1$ to ${\bar{R}}_{t=2}/{\bar{R}}_{t=0}=1.5$ (${\bar{R}}_{t}$: vesicle radius at time *t*) during the first 2 h and remained essentially constant during the 3 h pause (Fig. 1f). Upon feeding, the membrane area approximately doubled, indicating that nearly all of the supplied DA molecules were incorporated into the vesicle membranes. During the second supply, the normalized vesicle radius increased further from ${\bar{R}}_{t=5}/{\bar{R}}_{t=0}=1.5$ to ${\bar{R}}_{t=7}/{\bar{R}}_{t=0}=1.9$. When the feeding was stopped again, the vesicle size no longer changed (${\bar{R}}_{t=10}/{\bar{R}}_{t=0}=1.9$). In contrast, vesicles that were kept quiescent (without micelle supply) did not change their size during this 10 h period. The number of fatty acid molecules contained in a vesicle of radius at time *t* is given by ${N}_{t}=2\times 4\pi {\bar{R}}_{t}^{2}/{a}_{0}$, where ${a}_{0}$(= 0.226 nm^2^) is the cross-sectional area of a DA molecule^40,41^. Thus, we express the normalized average growth rate of the vesicles during the first DA micelle-supply period ($\Delta t=2\,h=7200\,{{\rm{s}}}$) as ${v}_{{{\rm{D}}}}=\frac{{N}_{t=2}^{{{\rm{D}}}}-{N}_{t=0}^{{{\rm{D}}}}}{{N}_{t=0}^{{{\rm{D}}}}\,\Delta t}\approx 1.9\times {10}^{-4}$ [s^-1^]. The normalized average growth rate over the total micelle-supply period (first + second supply: $2\Delta t=14400{{\rm{s}}}$) is ${v}_{{{\rm{D}}}}=\frac{{N}_{t=7}^{{{\rm{D}}}}-{N}_{t=0}^{{{\rm{D}}}}}{{N}_{t=0}^{{{\rm{D}}}}\,2\Delta t}\approx 1.9\times {10}^{-4}$ [s^-1^] showing good agreement between the two estimates. Therefore, the difference in vesicle area before and after 2 h feeding is used as an average vesicle growth rate. Importantly, no leakage of encapsulated calcein was detected from the DA vesicles, confirming the integrity of the vesicle membranes throughout the growth process (Supplementary Fig. 2).

### Growth of DA vesicles in the presence of amino acids

We investigate the effect of amino acid coexistence on the growth rate of DA vesicles using 16 amino acids and identify the key factors promoting vesicle growth. The 16 amino acids used in this study were Lys, His, Arg, Ser, Thr, Gln, Asn, Glu, Asp, Gly, Ala, Val, Leu, Pro, Ile, and Trp. Among them, Gly, Ala, Asp, Ser, and Glu are the amino acids frequently detected in carbonaceous chondrites^29,42^. The properties of the 16 amino acids, hydrophobicity^43^, dissociation constants for the carboxyl group (p*K*_1_) and the amino group (p*K*_2_)^44^, isoelectric point (pI)^45^ and molecular weight (MW), are summarized in Supplementary Table 1. A 100 mM DA solution containing 20 mM of one of the 16 amino acids was added over 2 hours, and the effect of the amino acid on the vesicle growth rate was estimated by DLS measurements. Typical intermediate scattering functions and size distribution functions in the presence of amino acids are shown in Supplementary Fig. 3. The mean hydrodynamic radius was obtained by the fitting the intermediate scattering function using Eq. (1). The normalized average vesicle growth rate by addition of DA + amino acid solution ($\Delta t=7200\,{{\rm{s}}}$) is given by ${v}_{{{\rm{D}}}+{{\rm{A}}}}=\frac{{N}_{t=2}^{{{\rm{D}}}+{{\rm{A}}}}-{N}_{t=0}^{{{\rm{D}}}+{{\rm{A}}}}}{{N}_{t=0}^{{{\rm{D}}}+{{\rm{A}}}}\,\Delta t}$. Note that ${N}_{t=2}^{{{\rm{D}}}+{{\rm{A}}}}$ and ${N}_{t=0}^{{{\rm{D}}}+{{\rm{A}}}}$ were measured in each DA vesicle growth experiment with each amino acid. Hereafter, the difference in the normalized growth rates,

2 $$p={v}_{{{\rm{D}}}+{{\rm{A}}}}-{v}_{{{\rm{D}}}}=\frac{1}{\Delta t}\left(\frac{{N}_{t=2}^{{{\rm{D}}}+{{\rm{A}}}}-{N}_{t=0}^{{{\rm{D}}}+{{\rm{A}}}}}{{N}_{t=0}^{{{\rm{D}}}+{{\rm{A}}}}\,}-\frac{{N}_{t=2}^{{{\rm{D}}}}-{N}_{t=0}^{{{\rm{D}}}}}{{N}_{t=0}^{{{\rm{D}}}}\,}\right)$$

is used as a quantitative fitness parameter. A positive *p* indicates that the amino acid promotes vesicle growth, while a negative *p* indicates that it suppresses vesicle growth. It should be noted that the fitness *p* is proportional to the change in the chemical potential of the DA molecule in the external solution due to its interaction with amino acids or peptides (see Eq. (7) in the section “Driving force for vesicle growth promoted by peptides”).

**Fig. 2:**
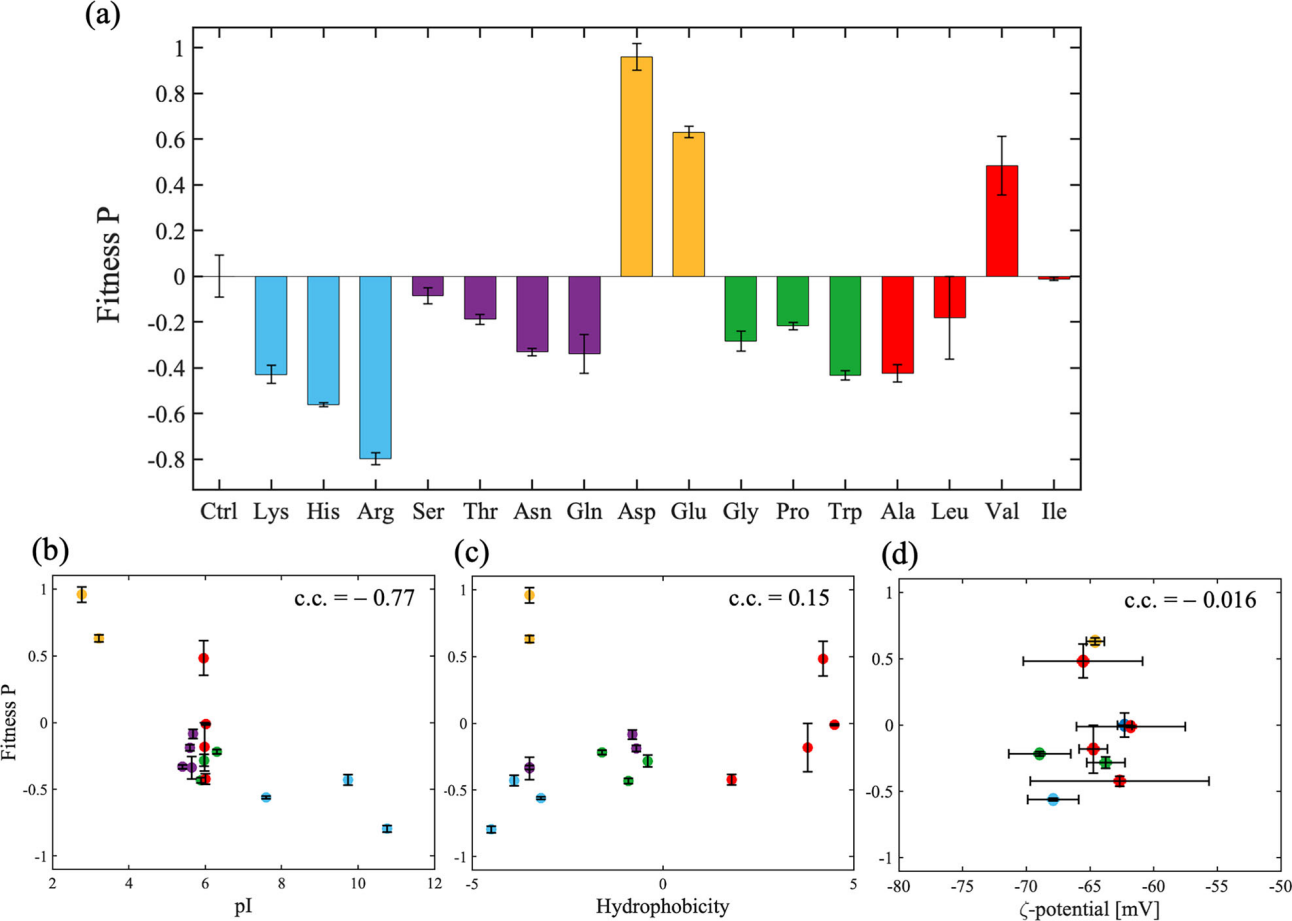
Growth of DA vesicles in the presence of amino acids. **a** Effect of amino acids on the average growth rate of DA vesicles (dimensionless fitness *P*). Ctrl indicates the addition of only DA molecules. Blue, violet, and orange bars indicate basic, neutral, and acidic polar amino acids, respectively. Green and red bars indicate nonpolar amino acids with weak and strong hydrophobicity, respectively. Positive *P* indicates promotion of vesicle growth, and negative *P* indicates suppression of vesicle growth. The error bars indicate SEM estimated from three to five different experiments. **b** Relationship between pI of amino acids and fitness, *P*, **c** Relationship between hydrophobicity of amino acids and fitness, **d** Relationship between *ζ*-potential of DA vesicles in the presence of amino acid and fitness. The color of the solid circle indicates the type of amino acid denoted in Fig. 2a. The correlation coefficient (C.C.) is indicated at the upper right corner of each figure. The error bars indicate SEM (see Supplementary Table 2).

The values of the dimensionless fitness parameter *P*(= $p\Delta t$ = 7200$p$) for 16 amino acids are shown in Fig. 2a and listed in Supplementary Table 2, where $P$ is directly proportional to the difference in the average growth rate in the presence and absence of the amino acid. The leftmost Ctrl in Fig. 2a corresponds to the fitness for addition of only DA molecules (without amino acid), *i.e*., *P* = 0. Basic amino acids (blue bars), Lys, His, and Arg, encourage deprotonation of DA molecules in the supply solution, resulting in negative *P* values, whereas acidic amino acids (orange bars), Glu and Asp, promote protonation, resulting in positive *P* values. Neutral amino acids (violet bars), Ser, Thr, Gln, and Asn, have negative *P* values between basic and acidic amino acids. The correlation coefficient between the pI of amino acids and fitness is −0.77 (Fig. 2b), indicating that the vesicle growth is promoted by amino acids that encourage the protonation of DA molecules. On the other hand, the hydrophobicity of amino acids is a parameter that measures their ability to adhere to fatty acid vesicle membranes^9,14,15^. Hydrophobic amino acids (red bars) have negative *P* values for Ala, and Leu, positive *P* value for Val, and neutral, *P* ~ 0, for Ile (hydrophobicity increases from left to right in Fig. 2a). Gly having hydrogen side chain, Pro having pyrrolidine side chain, and Trp having indole side chain are classified as non-polar amino acids with weak hydrophobicity denoted by green bars and have negative *P* values. The correlation coefficient between the hydrophobicity of amino acids and fitness is 0.15 (Fig. 2c), which indicates that the hydrophobicity does not play a significant role in DA vesicle growth. In addition, the surface potentials (*ζ*-potential) in the presence of amino acids were measured as a proxy for the adhesion of amino acids to the DA vesicle membrane surface. The *ζ*-potential of DA vesicles in the absence of amino acids was −62 mV. When amino acids were added, the *ζ*-potential varied in the range of −62 to −69 mV, but the effect of amino acids on the *ζ*-potential was not significant. The obtained *ζ* potentials are plotted against the fitness as shown in Fig. 2d, where the correlation coefficient between the *ζ* potential and fitness is −0.16. This result indicates that modulation of the negative surface potential by amino acids is not responsible for the vesicle growth. Correlation coefficients for other physical properties are summarized in Supplementary Table 1. Among the physicochemical properties examined, pI of the amino acids showed the strongest correlation with the fitness *P*, indicating that the amino acid−dependent modulation of vesicle growth is primarily driven by their effect on the protonation state of DA molecules.

Todd et al. examined the effect of amino acids and peptides on the growth rate of DA vesicles in HEPES buffer solution (pH ~7.6) using a FRET technique^14^. They showed that all examined amino acids (Ala, Asp, Glu, Gly, Leu, Pro, and Ser) did not promote vesicle growth, which is consistent with our results. Note that their DA vesicles were in a buffer solution, where protonation or deprotonation of DA by the amino acids is suppressed.

### Growth of DA vesicles in the presence of dipeptides

We examined the relationship between the amino acid sequence of dipeptides and fitness using 10 dipeptides composed of four amino acids: Leu (strongly hydrophobic), Gly (weakly hydrophobic), Glu (acidic), and His (basic). As in the case of amino acids, 20 mM dipeptides were added to the vesicle suspension together with DA molecules for 2 hours. In the peptide experiments, to minimize counterion effects we used salt-free peptides (see Materials section). Then, the DA vesicle suspension after supplying the peptides contained 100 mM DA, 10 mM peptide, and 100 mM NaCl. In addition, the pH range of the solutions after supplying the peptides was 7.3–7.7 for dipeptides. Accordingly, it should be noted that the peptide−vesicle suspensions used in the present experiments had nearly identical pH and ionic strength. Note that the only exception was HisHis, which required 16 mM acetate as a counterion to neutralize excess positive charge. However, the pH of the DA vesicle suspension after the addition of HisHis was 7.4, which is within the pH range of 7.3–7.7 for the addition of other peptides (Supplementary Table 3).

**Fig. 3:**
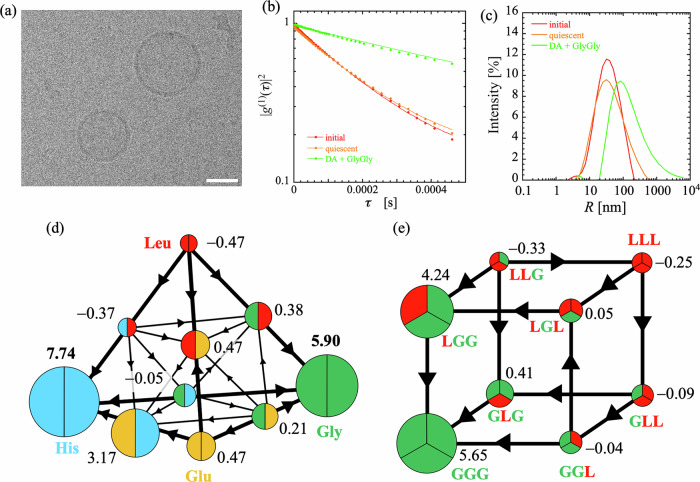
Growth of DA vesicles in the presence of peptides. **a** Cryo-TEM micrograph of DA vesicles just after the vesicle growth upon supplying DA micelles + dipeptide GlyGly (*t* = 2 h). Scale bar indicates 30 nm. **b** Intermediate scattering functions (at *q* = 2.22 $\times$ 10^7^ m^-1^) of DA SUVs at the initial state (*t* = 0 h), the quiescent state (*t* = 2 h) and after addition of 100 mM DA and 20 mM GlyGly solution (*t* = 2 h: DA + GlyGly). Solid lines indicate results of fitting using Eq. (1). **c** Size distribution functions for DA SUVs at the initial state (*t* = 0 h), the quiescent state (*t* = 2 h), and after the addition of DA + GlyGly solution (*t* = 2 h). The size distribution functions are obtained by inverse Laplace transformation of intermediate scattering functions using a constrained regularization program, CONTIN. A small additional peak at apparent size below ~5 nm observed in the sample with DA+GlyGly is not considered to represent vesicles but is attributed to small DA aggregates and/or noise in the DLS analysis. **d** Fitness landscape of dipeptides composed of Leu (red), Gly (green), Glu (orange), and His (blue) using a tetrahedral geometry. The dipeptide composition is represented by two half-circles filled with the color code. Each vertex represents a dipeptide composed of the same amino acid, and the midpoint of each edge is a dipeptide composed of amino acids at both ends (see text). The fitness of each peptide is visualized by the size of the circle. The value of *P* for each peptide is represented near the circle. The arrows point in the direction of amino acid sequences with higher fitness for a single replacement. **e** Fitness landscape of tripeptides composed of Leu (L) and Gly (G) using a cubic geometry. Each vertex represents a tripeptide with the sequence indicated by a circle. The tripeptide composition is represented by the color code in the circle counterclockwise from upper left, and indicated by letters L and G. The arrows point in the direction of amino acid sequences with higher fitness for a single replacement.

The cryo-TEM micrographs showed that after supplying DA molecules and dipeptide GlyGly, the DA vesicles kept their spherical and unilamellar structure (Fig. 3a) although they had large polydispersity. The vesicle size change was monitored by DLS. The intermediate scattering function for DA vesicles was drastically changed by supplying 100 mM DA molecules and 20 mM GlyGly (DA + GlyGly) for 2 h. as shown in Fig. 3b. The relaxation rate decreased by supplying DA + GlyGly, and DA vesicles increased their mean hydrodynamic radius from ${\bar{R}}_{t=0}^{{{\rm{DA}}}+{{\rm{GlyGly}}}}\approx 40$ nm to ${\bar{R}}_{t=2}^{{{\rm{DA}}}+{{\rm{GlyGly}}}}\approx 100$ nm, which means that in addition to the supplied DA molecules, the DA molecules that were initially present were also incorporated into the vesicle membrane. As the mean vesicle size grows, the size distribution shifts significantly toward larger vesicle size as shown in Fig. 3c. The normalized average vesicle growth rate by addition of DA + peptide solution (fixed $\Delta t=7200\,{{\rm{s}}}$) is given by ${v}_{{{\rm{D}}}+{{\rm{P}}}}=\frac{{N}_{t=2}^{{{\rm{D}}}+{{\rm{P}}}}-{N}_{t=0}^{{{\rm{D}}}+{{\rm{P}}}}}{{N}_{t=0}^{{{\rm{D}}}+{{\rm{P}}}}\,\Delta t}$. The fitness of DA vesicles in the presence of each dipeptide was quantified as $p={v}_{{{\rm{D}}}+{{\rm{P}}}}-{v}_{D}$ and the dimensionless fitness of dipeptide GlyGly is *P*_GlyGly_ = ${p}_{{{\rm{GlyGly}}}}\times \Delta t$ = 5.90, indicating that GlyGly significantly promotes vesicle growth. Intermediate scattering functions and size distribution functions of other typical dipeptide-fed DA SUV suspensions are shown in Supplementary Fig. 4.

The obtained fitness of each dipeptide is visualized by the fitness landscape, where fitness is mapped onto a tetrahedral geometry defined by peptide sequence, as shown in Fig. 3d and summarized in Supplementary Table 3. Each vertex represents a dipeptide composed of the same amino acid residues, and the midpoint of each edge is a dipeptide composed of amino acids at both ends. The dipeptide composition at each point is also represented by two half circles filled with a color code, Leu (red), Gly (green), Glu (orange), and His (blue), and the fitness of each dipeptide is denoted in numbers and visualized by the size of the circle. It should be noted that the dipeptides consisting of Leu and Gly have two patterns, Leu-Gly and Gly-Leu with *P*_LeuGly_ = 0.69±0.29 and *P*_GlyLeu_ = 0.38±0.08, respectively. For convenience, hereafter, we represent the fitness values of the six hetero-dipeptides using those of GlyLeu, LeuGlu, HisLeu, GlyGlu, GluHis, and GlyHis. The fitness landscape has two independent peaks at GlyGly (*P*_GlyGly_ = 5.90) and HisHis (*P*_HisHis_ = 7.74) separated by a deep valley along LeuLeu (*P*_LeuLeu_ = −0.47), HisLeu (*P*_HisLeu_ = −0.37), GlyHis (*P*_GlyHis_ = −0.05), and GlyGlu (*P*_GlyGlu_ = 0.21). Thus, Gly-Gly and His-His are the specific sequences promoting vesicle growth, which indicates the selective coupling between amino acid sequence and vesicle growth.

Here, we address the effect of pH on fitness. In the case of dipeptides, the growth rate (fitness) of the vesicles varies widely, from a maximum of *P*_HisHis_ = 7.74 to a minimum of *P*_LeuLeu_ = −0.47. Meanwhile, the pH of the DA vesicle suspension after peptide addition ranged only from 7.3 to 7.7 (Supplementary Table 3), and no correlation was observed between the fitness and solution pH. When comparing LeuLeu and GlyGly, the pH values of the peptide-micelle solutions before addition were 7.93 and 7.94, and the vesicle suspension after addition, they were 7.61 and 7.66, respectively, virtually identical. Nevertheless, the fitness values differed significantly: *P*_LeuLeu_ = −0.47 and *P*_GlyGly_ = 5.90, which clearly demonstrates that observed vesicle growth is promoted not by pH modulation and/or ionic strength of the solution but by the amino acid sequence of the peptide.

We examined how the individual amino acid residues influence the fitness of dipeptides. Since the effect of replacement of amino acid residue on the fitness depends not only on the type of the replaced residue but also on the type of adjacent residue, we quantified the contribution of each amino acid residue by averaging over all possible adjacent residues in the dipeptides. This approach corresponds to the schema average fitness as defined in prior studies^33–36^. For example, we focus on Leu. When Glu in the dipeptide Leu-Glu is replaced by Leu (yielding Leu-Leu), the change in the fitness is expressed by ∆*P*_20→00_ = *P*_00_ − *P*_20_ = −0.94 (Supplementary Table 4), where, for convenience, amino acids are represented as follows: Leu = 0, Gly = 1, Glu = 2, and His = 3, so that *P*_20_ refers to the fitness of Leu–Glu. Similarly, when the second residue (X) in Leu−X is replaced with Leu (X = Gly, Glu, or His), the average change in fitness is $\Delta {P}_{{{\rm{X}}}0\to 00}=\left[\left({P}_{00}-{P}_{10}\right)+\left({P}_{00}-{P}_{20}\right)+\left({P}_{00}-{P}_{30}\right)\right]/3=-0.63$ (averaging denoted by a subscript “X”). Assuming symmetry for hetero-dipeptides (*P*_AB_ = *P*_BA_, $A,{B}\in \left\{{{\rm{Leu}}},{{\rm{Gly}}},{{\rm{Glu}}},{{\rm{His}}}\right\}$), we also calculated the change in fitness when amino acid residue, X, is replaced by Leu in Gly–X, Glu–X, and His–X, yielding: ∆*P*_X1→01_ = −1.64, ∆*P*_X2→02_ = −0.81, and ∆*P*_X3→03_ = −4.00. The average contribution of Leu to dipeptide fitness is thus: $\Delta P \left({{\rm{Leu}}}\right) = \left[\Delta {P}_{{{\rm{X}}} 0 \to 00} + {\Delta P}_{{{\rm{X}}} 1 \to 01} + \Delta {P}_{{{\rm{X}}} 2 \to 02} + \Delta {P}_{{{\rm{X}}} 3 \to 03}\right]/4=-1.77$, indicating a net negative effect. Using the same approach, the average effects of Gly, Glu, and His on dipeptide fitness were found to be: $\Delta P\left({{\rm{Gly}}}\right)=0.38$, $\Delta P\left({{\rm{Glu}}}\right)=-0.47$, and $\Delta P\left({{\rm{His}}}\right)=1.73$. Interestingly, when compared to their effects as single amino acids (*P*_Leu_ = −0.18, *P*_Gly_ = −0.28, *P*_Glu_ = 0.63, and *P*_His_ = −0.56), Gly, Glu, and His exhibit opposite effects on vesicle fitness. These results indicate that the influence of an amino acid on vesicle growth is not conserved between its monomeric and dipeptide states and is strongly dependent on sequence context.

Next, we discuss whether the contribution of an amino acid residue to dipeptide fitness depends on the adjacent residue. As noted later (see “Driving force for vesicle growth promoted by peptides” section), the fitness is related to the chemical potential of the DA molecule in solution. If the contribution of the two residues in the dipeptides is independent, the fitness would be additive, and the fitness landscape would have a single global maximum. Epistasis refers to deviations from this independence^36^, often resulting in multiple local optima in the fitness landscape^23^. The observed dipeptide fitness landscape (Fig. 3d) shows two prominent peaks separated by a deep valley, clearly indicating epistatic interactions between residues. For example, consider the transitions along the HisHis (*P*_33_ = 7.74) $\leftrightarrow$ GlyHis (*P*_31_ = −0.05)$\,\leftrightarrow$ GlyGly (*P*_11_ = 5.90) path in Fig. 3d. Replacing the first His in HisHis with Gly causes a dramatic fitness drop ∆*P*_33→31_ = −7.79, whereas the same replacement in GlyHis leads to a large fitness increase ∆*P*_31→11_ = 5.95. Importantly, the sign of the fitness change reverses depending on the adjacent residue, demonstrating that the effect of an amino acid substitution is highly sequence-dependent. Similar sequence-dependent effects were observed for other residue combinations as well (Supplementary Table 4). These results demonstrate that the dipeptide fitness landscape constructed from Leu, Gly, Glu, and His is strongly epistatic. Furthermore, GlyGly has a high fitness of *P* = 5.90, while GlyLeu (*P* = 0.38), GlyHis (*P* = −0.05), and GlyGlu (*P* = 0.21) exhibit low fitness. Similarly, while HisHis exhibits a high fitness value of *P* = 7.74 and GluHis shows a relatively large value of *P* = 3.17, HisLeu (*P* = –0.37) and GlyHis (*P* = –0.05) exhibit negligible vesicle growth. Taken together, these results indicate that dipeptides with specific amino acid sequences significantly promote DA vesicle growth, analogous to sequence motifs in genetic polymers, and that the contribution of each residue to fitness is strongly influenced by its sequence context (epistasis).

### Growth of DA vesicles in the presence of tripeptides

Using tripeptides, we investigated whether the sequence-dependent effects on vesicle growth observed in dipeptides are preserved in longer peptides. In particular, we examined whether specific growth-promoting sequences retain their effects in tripeptides, and whether epistasis becomes more pronounced. To analyze sequence-dependent interactions in detail, we applied Walsh analysis, a mathematical method that decomposes the effects of sequence variations on fitness to quantify both individual and interaction effects among sequence elements^33,34,36^. We prepared tripeptides with 2^3^sequences consisting of Leu (0) and Gly (1). The fitness landscape, encompassing all possible combinations of Gly and Leu (a combinatorially complete set), was analyzed to uncover the effect of the amino acid residue replacement and to quantify the interactions between amino acid residues. As in the case of dipeptides, 20 mM tripeptides were added to the vesicle suspension together with DA molecules and the vesicle size was measured before and after the addition. Typical intermediate scattering functions and size distribution functions of DA SUV suspensions in the presence of the tripeptides are shown in Supplementary Fig. 5. The obtained fitness of each tripeptide is visualized by the fitness landscape using a cubic geometry as shown in Fig. 3e and summarized in Supplementary Table 5 along with the solution pH. Notably, for tripeptides, fitness values ranged from a maximum of *P* = 5.65 (GlyGlyGly) to a minimum of *P* = –0.33 (LeuLeuGly), while the pH of the vesicle suspensions after tripeptide addition was between 7.53 and 7.67. Again, no correlation was found between vesicle fitness and solution pH.

The fitness landscape of tripeptides composed of Leu (0) and Gly (1) displays a single global peak at GlyGlyGly (*P*_111_ = 5.65) and a minimum at LeuLeuGly (*P*_100_ = −0.33). Starting from the reference sequence LeuLeuLeu, there are four accessible monotonic paths leading to the peak, indicating a smooth gradient toward the optimal sequence. The GlyGly motif, which was previously identified as growth-promoting in dipeptides, appears in three tripeptides: GlyGlyGly, GlyGlyLeu, and LeuGlyGly, with fitness values of 5.65, –0.04, and 4.24, respectively. Among these, GlyGlyLeu does not promote vesicle growth, despite containing the same motif, suggesting that the position of the GlyGly sequence within the tripeptide strongly affects its function. These results indicate that the high fitness associated with the GlyGly motif is partially preserved in tripeptides, but its effect is modulated by adjacent residues.

To quantify the contribution of amino acid residue substitutions to tripeptide fitness, we performed a Walsh analysis of the fitness landscape. This analysis decomposes the fitness into components reflecting individual and higher-order interactions among sequence positions^33,34,36^. Specifically, we computed background-averaged epistatic coefficients (denoted by *ε*), where the fitness changes by amino acid residue replacements are averaged over all possible sequences for the remaining positions in the tripeptides. For example, the effect of replacing Leu with Gly at the first residue position (*i.e*., the third bit in the binary representation) was calculated by averaging fitness changes over four replacement patterns: 000 → 001, 010 → 011, 100 → 101, and 110 → 111. The background-averaged effect of this replacement, is given by ${\varepsilon }_{* * 1}=\left[\left({P}_{001}-{P}_{000}\right)+\left({P}_{\,011}-{P}_{010}\right)+\left({P}_{101}-{P}_{100}\right)+\left({P}_{111}-{P}_{110}\right)\right]/4=0.56$, where averaging is denoted by subscript “*”: * = 0 or 1. The averages of fitness values for single, pairwise and higher order replacements are expressed by the Walsh transform as linear combinations of fitness values. The transformation matrix for tripeptides is given by

3 $$\left(\begin{array}{c}{\varepsilon }_{* * * }\\ \begin{array}{c}{\varepsilon }_{* * 1}\\ {\varepsilon }_{* 1* }\\ \begin{array}{c}{\varepsilon }_{* 11}\\ {\varepsilon }_{1* * }\\ \begin{array}{c}{\varepsilon }_{1*1}\\ {\varepsilon}_{11*}\\ {\varepsilon}_{111}\end{array}\end{array}\end{array}\end{array}\right)={{\bf{V}}}{{\boldsymbol{* }}}\left(\begin{array}{cccccccc}1 & 1 & 1 & 1 & 1 & 1 & 1 & 1\\ 1 & -1 & 1 & -1 & 1 & -1 & 1 & -1\\ 1 & 1 & -1 & -1 & 1 & 1 & -1 & -1\\ 1 & -1 & -1 & 1 & 1 & -1 & -1 & 1\\ 1 & 1 & 1 & 1 & -1 & -1 & -1 & -1\\ 1 & -1 & 1 & -1 & -1 & 1 & -1 & 1\\ 1 & 1 & -1 & -1 & -1 & -1 & 1 & 1\\ 1 & -1 & -1 & 1 & -1 & 1 & 1 & -1\end{array}\right)* \left(\begin{array}{c}{P}_{000}\\ \begin{array}{c}{P}_{001}\\ {P}_{010}\\ \begin{array}{c}{P}_{011}\\ {P}_{100}\\ \begin{array}{c}{P}_{101}\\ {P}_{110}\\ {P}_{111}\end{array}\end{array}\end{array}\end{array}\right)$$

where **V** is a diagonal weighting matrix to account for averaging over different numbers of terms as a function of the order of epistasis; ${v}_{{ii}}={(-1)}^{{q}_{i}}/{2}^{3-{q}_{i}}$, where ${q}_{i}$ is the order of the epistatic contribution in row *i*. The calculated background-averaged values (Walsh coefficients) are listed in Supplementary Table 6. First, we consider the effect of a single amino acid residue replacement from Leu to Gly on the fitness (1st Walsh coefficient), where the replacements take place at three positions, **1, *1*, and 1**. If the effect of the replacement (Leu$\to$Gly) is independent of the remaining amino acid sequence, the three 1st Walsh coefficients have the same value. The background-averaged coefficients were computed for substitutions at the second (${\varepsilon }_{* 1* }$) and third (${\varepsilon }_{1* * }$) positions. The values obtained were: ${\varepsilon }_{* 1* }=2.54$, and ${\varepsilon }_{1* * }=2.57$. These results indicate that replacing Leu with Gly generally increases tripeptide fitness, but the magnitude of the effect strongly depends on the position within the sequence. Notably, the fitness increase at the first position is approximately one-fifth that observed at the second and third positions, highlighting the sequence dependence of residue effects.

Next, we examined second-order epistatic effects, corresponding to pairwise amino acid replacements from LeuLeu to GlyGly, using the second-order Walsh coefficients. These coefficients quantify the deviation from additivity when two residues are replaced simultaneously, compared to sequential single-residue substitutions. In an entirely additive system, the second-order coefficients should be zero. For example, we consider the case where the first and second residues are replaced. There are two replacement patterns for this case: 000 → 011 and 100 → 111. The second-order Walsh coefficient is calculated as: ${\varepsilon }_{* 11}=\left[\left\{{P}_{011}-\left({P}_{000}+\left({P}_{010}-{P}_{000}\right)+\left({P}_{001}-{P}_{000}\right)\right)\right\}+\left\{{P}_{111}-\left({P}_{100}+\left({P}_{110}-{P}_{100}\right)+\left({P}_{101}-{P}_{100}\right)\right)\right\}\right]/2=0.21$. This expression represents the difference between the observed fitness change for simultaneous replacement and the sum of fitness changes for individual replacements, averaged over both background sequences. We similarly calculated second-order coefficients for other position pairs: ${\varepsilon }_{1* 1}=1.05$ for first and third positions, and ${\varepsilon }_{11* }=4.73$ for second and third positions. These results show that pairwise Leu-to-Gly substitutions generally enhance fitness, but the magnitude of the epistatic effect is highly position-dependent. In particular, the second and third positions exhibit a strong synergistic effect, as indicated by the large value of ${\varepsilon }_{11* }$, whereas the interactions between the first and second (${\varepsilon }_{* 11}=0.21$) and between the first and third (${\varepsilon }_{1* 1}=1.05$) are relatively modest. These findings are consistent with the previously observed small first-order coefficient at the first position (${\varepsilon }_{* * 1}=0.56$). In summary, second-order epistasis reveals that pairwise amino acid replacements can synergistically enhance vesicle growth, and that the strength of this epistatic effect depends critically on the positions involved.

In all cases, the Walsh coefficients for tripeptides are positive, indicating that the Leu to Gly replacement increases the fitness (Supplementary Table 6). This is consistent with the dipeptide results of $\Delta P\left({{\rm{Gly}}}\right)=0.38$. The retention of the specific Gly-Gly sequence effect is supported by the high fitness of LeuGlyGly (*P*_110_ = 4.24) and GlyGlyGly (*P*_111_ = 5.65) sequences. Furthermore, a tripeptide with GlyGlyHis sequence, *P*_311_ = 7.99, and a tetrapeptide with GlyGlyGlyGly sequence, *P*_1111_ = 4.41, also show high fitness, suggesting that the advantage of the GlyGly sequence is retained in longer peptides. In this sense, our fatty acid vesicle–peptide protocells do not yet realize Darwinian evolution, but they already exhibit a structured, motif- and epistasis-based sequence–fitness landscape. Such a landscape is a basic requirement for generating diverse evolutionary responses, suggesting that simple peptide–vesicle assemblies can provide a physicochemical substrate that becomes evolvable once coupled to primitive replication processes.

Previous studies by Black’s group have reported that hydrophobic peptides, specifically dipeptide (LeuLeu) and tripeptide (LeuLeuLeu), promote the growth of fatty acid vesicles more effectively than GlyGly in 200 mM HEPES (4-(2-hydroxyethyl)-1-piperazineethanesulfonic acid) buffer solution^14,15^. Their molecular dynamics simulations suggested that the insertion of LeuLeu into the membrane is a key factor in vesicle growth^15,46^. In contrast, our experiments show that GlyGly has a high fitness value of *P* = 5.90, while LeuLeu suppresses vesicle growth with *P* = −0.47. As described above, we hypothesize that the specific interactions between DA molecules and peptides result in vesicle growth. One possible reason for the discrepancy is the presence of 200 mM HEPES in the previous study. The organic salt may have interfered with peptide−DA interactions, thereby altering vesicle growth behavior. Additionally, there is a procedural difference: in their experiments, peptides were added to the vesicle suspension prior to the addition of DA micelles, whereas in our protocol, peptides and DA micelles were added simultaneously. This difference in timing could also influence the interaction dynamics and the resulting vesicle growth. Therefore, in the next section, the driving force causing vesicle growth by peptides is examined from the perspective of the interactions between DA molecules and peptides.

### Driving force for vesicle growth promoted by peptides

To achieve vesicle growth by incorporating DA molecules in the external solution into the DA vesicle membrane, two conditions must be satisfied: 1) The chemical potential of a DA molecule in the external solution, ${\mu }_{{{\rm{ext}}}}$, must be larger than that in the membrane, ${\mu }_{{{\rm{mem}}}}$, causing the flux of DA molecules from the external solution to the membrane, and 2) The activation energy barrier for incorporation must be overcome with the thermal energy (Supplementary Fig. 6). The normalized growth rate of DA vesicles by incorporating DA molecules in the external solution is expressed by

4 $${v}_{{{\rm{D}}}}=\nu {e}^{-\Delta {u}_{{{\rm{a}}}}^{{{\rm{D}}}}/{k}_{{{\rm{B}}}}T}\left(1-{e}^{-\Delta {\mu }^{{{\rm{D}}}}/{k}_{{{\rm{B}}}}T}\right)$$

where the constant $\nu$ is proportional to the attempt frequency to overcome the energy barrier per second, $\Delta {u}_{{{\rm{a}}}}^{{{\rm{D}}}}$ is the activation energy per molecule to incorporate DA molecules into the membrane, and $\Delta {\mu }^{{{\rm{D}}}}={\mu }_{{{\rm{ext}}}}^{{{\rm{D}}}}-$ ${\mu }_{{{\rm{mem}}}}^{{{\rm{D}}}}$ is the difference in chemical potential of DA molecule in the external solution and in the vesicle membrane. For $\Delta {u}_{{{\rm{a}}}}^{{{\rm{D}}}}\ll {k}_{{{\rm{B}}}}T$, the vesicle growth is driven by the chemical potential difference $\Delta {\mu }^{{{\rm{D}}}}$ (thermodynamically driven), whereas for $\Delta {u}_{{{\rm{a}}}}^{{{\rm{D}}}}\gg {k}_{{{\rm{B}}}}T$, it is kinetically controlled.

In the present experiments, the vesicle suspension before the addition of the DA micellar solution is at equilibrium, *i.e*., ${\mu }_{{{\rm{ext}}}}^{{{\rm{D}}}}={\mu }_{{{\rm{mem}}}}^{{{\rm{D}}}}$. Accordingly, the concentration of DA molecules in the solution coincides with the CVC ( ~ 60 mM). To 2.0 mL of this vesicle suspension, 0.6 mL of a 100 mM DA micellar solution was added over 2 h at a rate of 4.8 μL/min while gently stirring. During this micelle supply, the mole fraction of DA molecules in the external aqueous phase, ${X}_{{{\rm{ext}}}}^{{{\rm{D}}}}$, increases only slightly compared with the equilibrium mole fraction ${X}_{{{\rm{CVC}}}}^{{{\rm{D}}}}$. However, the corresponding increase in chemical potential is given by ${k}_{{{\rm{B}}}}T{\mathrm{ln}}\left({X}_{{{\rm{ext}}}}^{{{\rm{D}}}}/{X}_{{{\rm{CVC}}}}^{{{\rm{D}}}}\right)$, which remains small in our protocol. As a conservative upper bound, simple dilution of 2.0 mL of a 60 mM DA vesicle suspension by 0.6 mL of a 100 mM DA micellar solution gives a final DA concentration of ~ 69 mM, corresponding to ${\mathrm{ln}}\left(69/60\right)\approx 0.14$, *i.e*., $\Delta {\mu }^{{{\rm{D}}}}\lesssim 0.14{k}_{{{\rm{B}}}}T$. Thus, during vesicle growth $\Delta {\mu }^{{{\rm{D}}}} < {k}_{{{\rm{B}}}}T$, and the normalized vesicle growth rate in this regime is expressed as

5 $${v}_{{{\rm{D}}}}\approx \nu {e}^{-\Delta {u}_{{{\rm{a}}}}^{{{\rm{D}}}}/{k}_{{{\rm{B}}}}T}\left(\Delta {\mu }^{{{\rm{D}}}}/{k}_{{{\rm{B}}}}T\right)\approx \nu {e}^{-\Delta {u}_{{{\rm{a}}}}^{{{\rm{D}}}}/{k}_{{{\rm{B}}}}T}{\mathrm{ln}}\left({X}_{{{\rm{ext}}}}^{{{\rm{D}}}}/{X}_{{{\rm{CVC}}}}^{{{\rm{D}}}}\right)$$

**Fig. 4:**
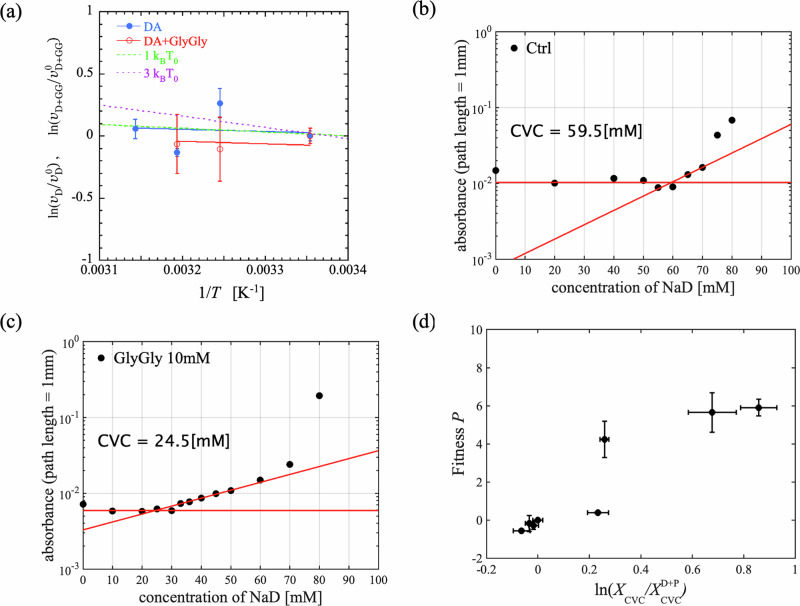
Driving force for vesicle growth promoted by peptides. **a** Arrhenius-type analysis of the apparent activation contribution for incorporation of DA molecules into the membrane in the absence and presence of GlyGly. The normalized average growth rates of DA vesicles, expressed as ${\mathrm{ln}}\left({v}_{{{\rm{D}}}}/{v}_{{{\rm{D}}}}^{0}\right)$ and ln$\left({v}_{{{\rm{D}}}+{{\rm{GG}}}}/{v}_{{{\rm{D}}}+{{\rm{GG}}}}^{0}\right)$, are plotted against 1/*T* for vesicle growth upon addition of 100 mM DA (blue circles, ctrl) and 100 mM DA + 20 mM GlyGly (red open circles, GlyGly), respectively, where ${v}_{{{\rm{D}}}}^{0}$ and ${v}_{{{\rm{D}}}+{{\rm{GG}}}}^{0}$ are the corresponding normalized growth rates at ${T}_{0}\,$= 298 K. Error bars indicate SEM from 3−5 independent measurements. Blue and red lines indicate reference trends based on Eqs. (5) and (6), respectively. For comparison, reference lines corresponding to $\Delta {u}_{a}=1\,{k}_{B}{T}_{0}$ and $3\,{k}_{B}{T}_{0}$ are also shown. **b** Dependence of absorbance (λ = 490 nm) of a DA solution on the concentration of sodium decanoate (NaD) at 20 °C. **c** Dependence of absorbance (λ = 490 nm) of a DA solution containing 10 mM GlyGly on the concentration of NaD at 20 °C. **d** Relationship between the dimensionless fitness *P* and the CVC, ${\mathrm{ln}}({X}_{{{\rm{CVC}}}}^{{{\rm{D}}}}/{X}_{{{\rm{CVC}}}}^{{{\rm{D}}}+{{\rm{P}}}})$. ${X}_{{{\rm{CVC}}}}^{{{\rm{D}}}}$ and ${X}_{{{\rm{CVC}}}}^{{{\rm{D}}}+{{\rm{P}}}}$ are the CVCs of DA molecules in the absence and presence of 10 mM amino acids or peptides, respectively. The vertical and horizontal error bars indicate SEM for *P* and ${\mathrm{ln}}({X}_{{{\rm{CVC}}}}^{{{\rm{D}}}}/{X}_{{{\rm{CVC}}}}^{{{\rm{D}}}+{{\rm{P}}}})$, respectively, estimated from three to five independent measurements.

We then assessed the apparent activation contribution using an Arrhenius-type plot of ${\mathrm{ln}}({v}_{{{\rm{D}}}}/{v}_{{{\rm{D}}}}^{0})$ versus 1/*T* (Fig. 4a), where ${v}_{{{\rm{D}}}}^{0}$ is the normalized growth rate at ${T}_{0}=$298 K. Although the DA data do not exhibit a strong straight-line trend, they are inconsistent with a strongly activated regime. This plot yields $\Delta {u}_{{{\rm{a}}}}^{{{\rm{D}}}}=0.3\pm 0.3\,{k}_{{{\rm{B}}}}{T}_{0}$, indicating that the activation contribution is small and not well resolved within experimental uncertainty.

Similarly, in the presence of peptides the external solution chemical potential of the DA molecule in solution (${\mu }_{{{\rm{ext}}}}^{{{\rm{D}}}+{{\rm{P}}}}$) and the apparent activation contribution ($\Delta {u}_{{{\rm{a}}}}^{{{\rm{D}}}+{{\rm{P}}}}$) may change, and in the same small-$\Delta \mu$ regime the growth rate can be written as

6 $${v}_{{{\rm{D}}}+{{\rm{P}}}}\approx \nu {e}^{-\Delta {u}_{{{\rm{a}}}}^{{{\rm{D}}}+{{\rm{P}}}}/{k}_{{{\rm{B}}}}T}\left(\Delta {\mu }^{{{\rm{D}}}+{{\rm{P}}}}/{k}_{{{\rm{B}}}}T\right)$$

where $\Delta {\mu }^{{{\rm{D}}}+{{\rm{P}}}}={\mu }_{{{\rm{ext}}}}^{{{\rm{D}}}+{{\rm{P}}}}-{\mu }_{{{\rm{mem}}}}^{{{\rm{D}}}}$ and we assume $\nu$ remains unchanged. In Fig. 4a, the temperature dependence of ${\mathrm{ln}}({v}_{{{\rm{D}}}+{{\rm{GG}}}}/{v}_{{{\rm{D}}}+{{\rm{GG}}}}^{0})$ for a representative growth-promoting peptide (GlyGly) is also shown, and the corresponding apparent activation contribution is indistinguishable from $\Delta {u}_{{{\rm{a}}}}^{D}$ within experimental uncertainty ($\Delta {u}_{{{\rm{a}}}}^{{{\rm{D}}}+{{\rm{GG}}}}\approx \Delta {u}_{{{\rm{a}}}}^{{{\rm{D}}}}$). These results suggest that peptide-enhanced growth is not due to a detectable reduction of the activation barrier, but is instead consistent with an increased driving chemical-potential difference. Therefore, the effect of peptides on the vesicle growth rate, *i.e*., fitness *p*, is expressed by

7 $$p={v}_{{{\rm{D}}}+{{\rm{P}}}}-{v}_{{{\rm{D}}}}\approx v{e}^{-\Delta {u}_{{{\rm{a}}}}^{{{\rm{D}}}}/{k}_{{{\rm{B}}}}T}\left[\frac{\Delta {\mu }^{{{\rm{D}}}+{{\rm{P}}}}-\Delta {\mu }^{{{\rm{D}}}}}{{k}_{{{\rm{B}}}}T}\right]=v{e}^{-\Delta {u}_{{{\rm{a}}}}^{{{\rm{D}}}}/{k}_{{{\rm{B}}}}T}\frac{\Delta {\mu }_{{{\rm{ext}}}}^{{{\rm{P}}}}}{{k}_{B}T}$$

This indicates that the fitness, *p* is proportional to the change in chemical potential of DA unimer in the external solution, $\Delta {\mu }_{{{\rm{ext}}}}^{{{\rm{P}}}}={\mu }_{{{\rm{ext}}}}^{{{\rm{D}}}+{{\rm{P}}}}-{\mu }_{{{\rm{ext}}}}^{{{\rm{D}}}}$, due to modification of the interaction energy by peptide binding. The chemical potentials of DA molecules in the external solution, ${\mu }_{{{\rm{ext}}}}^{{{\rm{D}}}}$ and ${\mu }_{{{\rm{ext}}}}^{{{\rm{D}}}+{{\rm{P}}}}$, are given by

8 $${\mu }_{{{\rm{ext}}}}^{{{\rm{D}}}}\approx {\mu }_{{{\rm{ext}}}}^{0({{\rm{D}}})}+{k}_{{{\rm{B}}}}T{\mathrm{ln}}\left({X}_{{{\rm{ext}}}}^{{{\rm{D}}}}\right)$$

9 $${\mu }_{{{\rm{ext}}}}^{{{\rm{D}}}+{{\rm{P}}}}\approx {\mu }_{{{\rm{ext}}}}^{0({{\rm{D}}}+{{\rm{P}}})}+{k}_{{{\rm{B}}}}T{\mathrm{ln}}\left({X}_{{{\rm{ext}}}}^{{{\rm{D}}}+{{\rm{P}}}}\right)$$

where ${\mu }_{{{\rm{ext}}}}^{0({{\rm{D}}})}$ and ${\mu }_{{{\rm{ext}}}}^{0({{\rm{D}}}+{{\rm{P}}})}$ are the standard chemical potentials of DA molecule in the absence and presence of peptides, respectively, and ${X}_{{{\rm{ext}}}}^{{{\rm{D}}}+{{\rm{P}}}}$ is the mole fraction of DA molecules in the external solution when DA micelles and peptides are supplied from outside to a decanoate solution that is at equilibrium, ${X}_{{{\rm{CVC}}}}^{{{\rm{D}}}}$. Thus,

10 $$\Delta {\mu }_{{{\rm{ext}}}}^{{{\rm{P}}}}={\mu }_{{{\rm{ext}}}}^{0({{\rm{D}}}+{{\rm{P}}})}-{\mu }_{{{\rm{ext}}}}^{0({{\rm{D}}})}+{k}_{{{\rm{B}}}}T\,{\mathrm{ln}}\left({X}_{{{\rm{ext}}}}^{{{\rm{D}}}+{{\rm{P}}}}/{X}_{{{\rm{ext}}}}^{{{\rm{D}}}}\right)$$

On the other hand, at equilibrium (where the mole fraction of DA molecules in the external solution is at the CVC), the condition ${\mu }_{{{\rm{ext}}}}^{{{\rm{D}}}}={\mu }_{{{\rm{mem}}}}^{{{\rm{D}}}}$ holds, which leads to

11 $${X}_{{{\rm{CVC}}}}^{{{\rm{D}}}}=\exp \left[-\left({\mu }_{{{\rm{ext}}}}^{0({{\rm{D}}})}-{\mu }_{{{\rm{mem}}}}^{0({{\rm{D}}})}\right)/{k}_{B}T\right]$$

where ${\mu }_{{{\rm{mem}}}}^{0({{\rm{D}}})}$ is the standard chemical potential of DA molecule in the vesicle membrane^47^. Similarly, the CVC in the presence of peptide is given by

12 $${X}_{{{\rm{CVC}}}}^{{{\rm{D}}}+{{\rm{P}}}}=\exp \left[-\left({\mu }_{{{\rm{ext}}}}^{0({{\rm{D}}}+{{\rm{P}}})}-{\mu }_{{{\rm{mem}}}}^{0({{\rm{D}}})}\right)/{k}_{B}T\right]$$

Here, we assume that DA molecules do not interact with the peptide in the membrane, so that their standard chemical potential in the membrane is essentially unchanged. It then follows that

13 $${\mu }_{{{\rm{ext}}}}^{0({{\rm{D}}}+{{\rm{P}}})}-{\mu }_{{{\rm{ext}}}}^{0({{\rm{D}}})}={k}_{B}T{\mathrm{ln}}\frac{{X}_{{{\rm{CVC}}}}^{{{\rm{D}}}}}{{X}_{{{\rm{CVC}}}}^{{{\rm{D}}}+{{\rm{P}}}}}$$

Thus, we obtain

14 $$p\approx v{e}^{-\Delta {u}_{{{\rm{a}}}}^{{{\rm{D}}}}/{k}_{{{\rm{B}}}}T}\left[{\mathrm{ln}}\frac{{X}_{{{\rm{CVC}}}}^{{{\rm{D}}}}}{{X}_{{{\rm{CVC}}}}^{{{\rm{D}}}+{{\rm{P}}}}}+{\mathrm{ln}}\frac{{X}_{{{\rm{ext}}}}^{{{\rm{D}}}+{{\rm{P}}}}}{{X}_{{{\rm{ext}}}}^{{{\rm{D}}}}}\right]$$

In our experiments, ${X}_{{{\rm{ext}}}}^{{{\rm{D}}}}$ and ${X}_{{{\rm{ext}}}}^{{{\rm{D}}}+{{\rm{P}}}}$ are both only slightly higher than ${X}_{{{\rm{CVC}}}}^{{{\rm{D}}}}$, and the difference between them is very small because the volume and concentration of the added micellar solution are limited. Therefore, ${\mathrm{ln}}\left({X}_{{{\rm{ext}}}}^{{{\rm{D}}}+{{\rm{P}}}}/{X}_{{{\rm{ext}}}}^{{{\rm{D}}}}\right)\approx 0$, which leads to

15 $$p\approx v{e}^{-\Delta {u}_{{{\rm{a}}}}^{{{\rm{D}}}}/{k}_{{{\rm{B}}}}T}{\mathrm{ln}}\frac{{X}_{{{\rm{CVC}}}}^{{{\rm{D}}}}}{{X}_{{{\rm{CVC}}}}^{{{\rm{D}}}+{{\rm{P}}}}}$$

To demonstrate the relationship between the fitness and the peptide-induced change in the chemical potential, $\Delta {\mu }_{{{\rm{ext}}}}^{{{\rm{P}}}}$, we estimated the CVC of DA vesicle suspensions and DA vesicle + peptide suspensions. Fig. 4b shows a representative example of the dependence of absorbance (λ = 490 nm) of a DA solution on sodium decanoate (NaD) concentration at 20 °C and pH $\approx$ 7.8. The absorbance remained constant up to ~60 mM and then began to increase due to the formation of DA vesicles. The CVC was estimated from the intersection of the linear baseline and the initial slope as ${C}_{{{\rm{CVC}}}}^{{{\rm{D}}}}=$ 59.5 mM in molar concentration, *i.e*., ${X}_{{{\rm{CVC}}}}^{{{\rm{D}}}}=1.0\,\times {10}^{-3}$. In the presence of dipeptide GlyGly with *P* = 5.9, the measured CVC was ${C}_{{{\rm{CVC}}}}^{{{\rm{D}}}+{{\rm{GG}}}}=$ 24.5 mM (${X}_{{{\rm{CVC}}}}^{{{\rm{D}}}+{{\rm{GG}}}}=4.4\times {10}^{-4}$), indicating the interaction between DA molecules and GlyGly in the external solution destabilizes the DA unimer state, ${\mu }_{{{\rm{ext}}}}^{0({{\rm{D}}}+{{\rm{GG}}})}={\mu }_{{{\rm{ext}}}}^{0({{\rm{D}}})}+0.9{k}_{B}T$, and promotes the incorporation into DA vesicles (the corresponding turbidity profile for GlyGly is shown in Fig. 4c). In this study, CVC measurements were carried out for three amino acids, two dipeptides, and two tripeptides. Turbidity profiles for three amino acids (His, Leu, and Gly) and three peptides (GlyLeu, LeuGlyGly, and GlyGlyGly) are shown in Supplementary Fig. 7. The obtained CVC values in the presence of amino acids and peptides are listed in Supplementary Table 7, together with their fitness values *P*. Note that the CVC values in the absence and presence of amino acids or peptides were obtained by averaging over three to five independent experiments. To examine the relationship between fitness and the change in the interaction energy, Fig. 4d plots the fitness *P* as a function of the estimated ${\mathrm{ln}}\,({X}_{{{\rm{CVC}}}}^{{{\rm{D}}}}/{X}_{{{\rm{CVC}}}}^{{{\rm{D}}}+{{\rm{P}}}})$. The data show a strong positive correlation in the range $0\lesssim {\mathrm{ln}}\,({X}_{{{\rm{CVC}}}}^{{{\rm{D}}}}/{X}_{{{\rm{CVC}}}}^{{{\rm{D}}}+{{\rm{P}}}})\lesssim 1$. In other words, vesicle growth is more strongly promoted when the interaction with peptides results in a greater reduction in CVC, indicating that modulation of the chemical potential by peptides is responsible for the vesicle growth promotion.

When DA molecules and peptides are supplied to DA vesicle suspensions, decanoate molecules in the external solution interact with peptides through the DA carboxyl group and the peptide amino group. This interaction reduces the effective hydrophilicity of DA in the external phase, thereby increasing the thermodynamic driving force for DA incorporation into the vesicle membrane. Vesicle growth coupled with peptides is therefore interpreted primarily as a thermodynamically driven process.

More specifically, peptides in the external solution lower the CVC of DA and thereby increase the driving chemical-potential difference for DA incorporation by shifting the external-phase chemical potential of DA from ${\mu }_{{{\rm{ext}}}}^{{{\rm{D}}}}$ to ${\mu }_{{{\rm{ext}}}}^{{{\rm{D}}}+P}$, thus increasing $\Delta {\mu }^{{{\rm{D}}}+{{\rm{P}}}}={\mu }_{{{\rm{ext}}}}^{{{\rm{D}}}+{{\rm{P}}}}-{\mu }_{{{\rm{mem}}}}^{{{\rm{D}}}}$. This bulk effect is the primary mechanism supported by our experimental data. Because DA incorporation occurs at the membrane interface, membrane-associated peptides (although they represent only a small fraction of the total peptide population on the vesicle surface^12^) may also contribute at the point of incorporation. In the present two-state framework, such effects are incorporated phenomenologically through an apparent local chemical potential of DA in the external solution near the membrane, ${\mu }_{{{\rm{ext}}}}^{{{\rm{D}}}+{{\rm{P}}}}$ (and thus $\Delta {\mu }^{{{\rm{D}}}+{{\rm{P}}}}$). Accordingly, vesicles associated with peptide sequences that yield larger $\Delta {\mu }^{{{\rm{D}}}+{{\rm{P}}}}$ are expected to grow faster and to be favored under competition among fatty-acid vesicle–peptide protocells.

The role of the present model is to provide a minimal decomposition of the growth rate into an activation contribution and a thermodynamic driving contribution, while the experiments identify which of these two terms is predominantly modulated by peptide sequence. The Arrhenius-type analysis indicates that the activation contribution is small and not detectably altered by peptide addition within experimental uncertainty, whereas the measured peptide-dependent CVC shifts provide an experimental proxy for changes in the driving force. Together, these results indicate that peptide-sequence-dependent growth is governed primarily by thermodynamic modulation of the driving chemical-potential difference rather than by changes in the activation barrier. The model therefore makes a testable prediction: peptide sequences that lower the CVC should systematically promote faster vesicle growth, provided that the activation contribution remains small. In some cases, the observed vesicle growth exceeded the amount expected from the supplied DA alone. This can be understood as a consequence of peptide-induced CVC reduction, which shifts the equilibrium so that additional pre-existing DA unimers in the external solution are incorporated into the vesicle membranes.

## Conclusions

In this study, we demonstrated that the growth of fatty acid vesicles is significantly promoted by specific primordial peptides, in a manner dependent on their amino acid sequences. Such selectivity arises from the modulation of the chemical potential of fatty acids by peptides and occurs spontaneously due to thermodynamic favorability. Furthermore, the peptide-fatty acid vesicle-based protocells exhibit the following features: 1) Peptides with specific amino acid sequences promote vesicle growth, analogous to sequence motifs in modern proteins; 2) The vesicle growth motif in peptides is largely retained as peptide length increases; and 3) the system exhibits epistasis, thereby generating protocell diversity. The observed characteristics suggest that peptide-fatty acid vesicle-based protocells encode non-trivial sequence−fitness landscapes for primitive compartment growth.

These findings highlight a mechanism by which molecular sequence information can be coupled to compartment growth in the absence of any biological machinery. Many protocell models hypothesize that the protometabolic reaction networks eventually synthesize membrane molecules in a manner dependent on the sequence of genetic polymers, but how such tightly regulated synthetic pathways could first arise remains unclear. By contrast, our results show that sequence-dependent peptide−fatty-acid interactions alone are sufficient to bias vesicle growth, without requiring dedicated biosynthetic pathways. Such thermodynamically driven coupling between peptide sequence motifs and protocell growth provides a plausible physical basis that could later be integrated with primitive replication processes on the path toward evolvable cellular systems.

## Methods

### Materials

Sodium Decanoate (purity: >99.0%) was obtained from Tokyo Chemical Industry (Tokyo, Japan) and used without further purification. The hydrochloric acid and sodium hydroxide solutions used for pH adjustment were prepared by diluting 6 M hydrochloric acid and 5 M sodium hydroxide purchased from Wako Pure Chemical Industries (Osaka, Japan). Ultrapure water purified with a Direct-Q 3 UV apparatus (Millipore, USA) was used to prepare all aqueous solutions and suspensions.

The 16 amino acids used in the experiments were purchased from Wako Pure Chemical Industries (Osaka, Japan) as listed in Supplementary Table 8 and stored at room temperature, except for Lys, which was stored at 5 °C. The suppliers, purities, and storage temperatures of the peptides used in this study are summarized in Supplementary Table 9. All amino acids and peptides used in this study were in the L configuration. We paid special attention to using salt-free zwitterionic peptides, since counterions of peptides may affect the growth rate of the vesicles. The counterions of the peptides used were evaluated by elemental analysis (Combustion: YHS-11, Yanaco, Japan, and Ion chromatography: IC-8100ST, Tosoh Corporation, Japan), and it was confirmed that F, Cl, Br, I, and S were all below the detection limits (F: 0.0056%, Cl: 0.0104%, Br: 0.032%, I: 0.128%, and S: 0.0128%). The purity of the peptides was confirmed to be greater than 98%, except for LeuLeuLeu, which was over 90%. It should be noted that when peptides are synthesized using the Fmoc solid-phase synthesis method, trifluoroacetic acid (TFA) is used to cleave the peptide from the resin, and as a result, TFA is known to remain as a counterion in the peptide products. Salt-free peptides were prepared by removing TFA using the ion exchange resin. We confirmed by the elemental analysis that the resulting peptides were free of salt. The only exception was HisHis, which required 16 mM acetate as a counterion to neutralize excess positive charge. However, the pH of the vesicle suspension after the addition of HisHis was 7.4, well within the range observed for the other peptides (pH 7.3−7.7; see Supplementary Table 3). These amino acids and peptides were used without further purification.

### Preparation of fatty acid vesicles and micellar solution

Since formation of fatty acid vesicles is restricted to pH range of 6–8, usually DA vesicles are prepared by adjusting the pH using buffer solutions^31^. The buffer solutions, however, reduce influences of pH on vesicle growth significantly. We therefore prepared DA vesicles without buffer solutions, applying the pH titration method^48^.

To prepare the vesicle suspension, 200.0 mg of sodium decanoate was dissolved in 9.8 mL of ultrapure water at 25 °C to yield a 100 mM sodium decanoate micellar solution. This micellar solution was sonicated for 10 minutes using an ultrasonic bath AU-12C (Aiwa Medical Industry, Japan). The micellar solution was titrated with 235 μL of 1 M HCl solution using a pipette (25 °C) and then mixed by vortex mixer MF-71 (Tokyo Garasu Kikai, Japan) to form DA giant vesicle (GV) suspension (final pH 7.7). The resulting GV suspension was allowed to stabilize for 24 hours at room temperature.

To prepare SUVs, 4 mL of the vesicle suspension was transferred to a 10 mL glass vial and sonicated at 6 W power using a Branson Sonifier ultrasonic cell disruptor/homogenizer 150-D with 23 kHz frequency (Branson, U.S.A.) for 40 s twice (interval time was 20 s). Each 2.0 mL of the SUV suspension was transferred to two 5.0 mL Eppendorf Tubes (Eppendorf, Germany) and centrifuged at 2500 rpm for 10 min using a centrifuge HIMIC CR5B2 (Hitachi Koki, Japan) to remove trace amounts of dissolved iron from the tip of the sonicator. The supernatant solution was filtered through a 200 nm membrane filter 6786-2502 (Cytiva, Japan) to remove dust present in the vesicle suspension. The obtained DA SUV suspension was stored at room temperature and used within 24 h after preparation.

A micellar solution was prepared as follows. Sodium decanoate was dissolved in ultra-pure water to a final concentration of 100 mM at approximately pH 9.5 (25 °C). This micellar solution was sonicated for 10 minutes using the ultrasonic bath and left at room temperature for 1 day; no longer than 2 days. Before using the micellar solution, the solution was filtered through the 200 nm membrane filter. When amino acids or peptides were supplied to a vesicle suspension, they were dissolved in the micellar solution to a concentration of 20 mM. Then, the mixed solution was sonicated for 10 minutes using the ultrasonic bath.

### Fatty acid vesicle growth experiments

In this study, average vesicle growth rates were estimated by measuring mean vesicle size before and after the addition of only DA micellar solution (Ctrl) and DA + amino acid or peptide mixed solution. During the injection of the DA micellar solution (pH ~9.5) into the parent DA SUV suspension (pH 7.7), it was important to maintain a constant pH in the suspension to avoid growth artifacts caused by pH perturbations. To achieve this, the DA micellar solution and HCl solution were slowly injected simultaneously over 2 hours^49^ using syringe pumps (YSP-101 standard type and YSP-2022 syringe-mounted type, YMC, Japan). The DA + amino acid or peptide mixture was prepared and injected in the same manner as the control.

A 0.7 mL aliquot of SUV suspension was transferred to a 2 mL vial. The lid of the 2-mL vial had two holes for injection syringes and one air hole. 0.1 mL of 0.1 M hydrochloric acid solution (pH~2) and 0.6 mL of the injection solution were simultaneously added with the injection rate of 0.8 μL/min and 4.8 μL/min, respectively, and the injection was performed over 2 h. For the injection syringe, a polytetrafluoroethylene (PTFE) nozzle with a 70 mm nozzle length TN-22G (Musashi Engineering, Japan) was attached to the tip of 1 mL syringe without a needle SS-01T (Terumo, Japan). During injection, the mother suspension was stirred at 600 rpm using a magnetic stirrer MA100 (Yamato Scientific, Japan) with a micro-rotor (5 × 2 mm *ϕ*) (AS ONE, Japan).

DLS and pH measurements were performed for the mother suspension before and after injection of the mixed solution. For control experiments (quiescent DA vesicle state), we measured SUV size without injection of the mixture solution.

### Direct observation of fatty acid vesicle growth using a microinjection technique

A target GV was held by the holding pipette Piezo Drill Tip with an inner diameter of $6\pm 0.5$ μm (Eppendorf, Germany). The suction pressure was kept as low as possible to hold the GV with the microinjector Cell Tram Vario (Eppendorf, Germany). A hand-made injection pipette (inner tip diameter: $2\pm 0.5$ μm), pulled using a micropipette puller PC-100 (Narishige, Japan), was filled with 100 mM DA micellar solution and mounted on the hydraulic micromanipulator MMO-202ND and MN-4 (Narishige, Japan). The pipette tip was positioned in front of the target vesicle at a distance of ~20 μm. The micellar solution was then ejected toward the target vesicle using a microinjection system FemtoJet (Eppendorf, Germany) at a constant injection pressure (20−60 hPa) during each experiment. Once the injection was initiated, the vesicle began to grow, and the average vesicle growth rate was quantified from the time evolution of the membrane area, which was obtained geometrically from the displacement of the end of the vesicle moving inside the holding capillary, as shown in the Supplementary Fig. 1.

### pH measurement

A tabletop pH meter and a Micro ToupH electrode 9618S-10D (HORIBA, Japan) were used for pH measurement. For pH measurements a set of pH standard buffer solutions ( ± 0.02 pH) 101-S (HORIBA, Japan) was used to calibrate the pH meter at three points, pH 4, 7, and 9.

It is known that the local pH at the surface of fatty acid membranes may differ from the bulk pH due to counterion condensation at the membrane interface^50^. Nevertheless, previous studies have shown that the protonation and ionization behavior of fatty acid vesicles can be reliably characterized using bulk pH values measured with this calibrated method^30,31^.

### Dynamic light scattering measurement

The dynamic light scattering (DLS) measurements were performed using an ALV-5000 goniometer system with an ALV-6000 multi-bit multi-tau correlator (ALV, Germany), and a diode-pumped laser Verdi V-2 (Coherent, U.S.A.) operating with vertically polarized light at a wavelength of 532 nm. The intermediate scattering functions were obtained from measurements with a duration time of 30 s and accumulation of 3 runs. The temperature was fixed at 25 ( ± 0.1) °C using a thermostat bath. The quartz cells for the DLS measurements were dipped in phosphorus-free detergent and ultrapure water, respectively, and then they were cleaned in an ultrasonic bath. After drying in a thermostatic bath at 40 °C, they were cleaned in an ultrasonic bath with ethanol for 10 min and dried in the thermostatic bath at 40 °C. Finally, the cells were thoroughly washed with 200 nm filtered acetone and then dried. This process was done to minimize dust inside the cells. Measurements were performed at five scattering angles: 60°, 80°, 90°, 100°, and 120°. The mean hydrodynamic radius of SUVs, $\bar{R}$, obtained from each scattering angle was averaged and used in the vesicle growth rate analysis. The size distribution of the vesicles is obtained by inverse Laplace transform (CONTIN analysis^38^) of the intermediate scattering function expressed in Eq. (1).

Here, we address the size polydispersity of SUVs obtained by the DLS. The CONTIN analysis of the intermediate scattering functions revealed that the SUVs exhibit substantial size polydispersity. When vesicles are grown significantly by supplying DA and peptides, the size distribution function shifts toward larger sizes and becomes broader (Fig. 3c, Supplementary Figs. 4b. 5b, f). In contrast, when vesicle growth is minimal, the size distribution function remains largely unchanged (Supplementary Figs. 4d and 5d). These results indicate that, despite the intrinsic polydispersity of SUVs, DLS measurements are capable of clearly detecting vesicle growth under the experimental conditions used. Therefore, we considered that the mean hydrodynamic radius obtained from DLS measurements could be used to evaluate the fitness, although it includes uncertainty due to the size distribution.

### Cryo-TEM observation

A few μL aliquots of the sample solutions were applied to a holey carbon film, Quantifoil R1.2/1.3 (Quantifoil Micro Tools, Germany) over a copper grid with 200 meshes at 25 °C. The grids were blotted with a filter paper and plunged into liquid ethane using a semi-automatic EM GP2 plunger (Leica Microsystems, Germany). The frozen grids were transferred to a CRYO ARM 300II electron microscope (JEOL, Japan) equipped with a cold-field emission gun operated at 300 kV. The images were recorded with an in-column energy filter with a slit width of 20 eV and at a nominal magnification of $\times$ 25,000 on a Gatan K3 direct electron detector (AMETEK, U.S.A.). The nominal defocus range was −2 μm. Each image stack consisting of 50 dose-fractionated frames was exposed at a dose rate of 2.8 to 18.1 e^−^ Å^−2^ sec^−1^ for 4 s in CDS mode and the resultant frames were aligned and summed using DigitalMicrograph (AMETEK, U.S.A.).

### *ζ*-potential measurement

As a measure of the surface potential, we measured *ζ*-potential that is the electrostatic potential at the slipping plane in the electric double layer. For *ζ*-potential measurements, we used SUVs of 100 mM DA in the absence and presence of 10 mM amino acids or peptides prepared by the fatty acid vesicle growth method. *ζ*-potentials of vesicles were measured at 25 °C using a *ζ*-potential analyzer, ELSZ-2000 (Otsuka Electronics, Japan). The SUV suspension was loaded in the sample cell, EZ2-870 (Otsuka Electronics, Japan) for the measurement. We carried out the measurement three times for each sample and the obtained electrophoretic mobility, *ξ*, was converted to *ζ*-potential, *ζ*, using the Helmholtz-Smoluchowski equation^51^.

### Turbidity measurement

Turbidity of NaD solution was measured at 20 °C using a V-730 spectrometer (JASCO, Japan) with quartz cuvettes S15-UV-1 (GL Sciences, Japan) having an optical path length of *L* = 0.1 cm. The cell was washed five times each with ultra-pure water and ethanol and then dried by blowing nitrogen gas to remove ethanol from inside and outside of the cell. Absorption spectrum was obtained for each sample in the region of UV/vis/NIR (190–1100 nm). After subtraction of the baseline, absorbance at 490 nm was used to determine the CVC.

### CVC measurement

For CVC measurements, we measured the turbidity of DA solution in the absence and presence of amino acid or peptide as a function of the concentration of NaD. Since the CVC of DA depends on the pH, first we adjusted the pH of each NaD solution to ~7.8 using hydrochloric acid, as shown in Supplementary Table 10. After the pH adjustment, 10 mM of amino acid or peptide was dissolved in the NaD solution. The samples were homogenized in an ultrasonic bath for 10 min and left at room temperature for 1 day. Before the turbidity measurement, we checked the pH of the solution. To equilibrate the sample, the sample was left in the cell chamber at 20 °C for 14 min and the absorption spectrum was measured. The obtained absorbance was plotted against the concentration of NaD (Figs. 4b, and 4c, and Supplementary Fig. 7). The turbidity profile is described by two lines, one horizontal line indicating no vesicle formation and the other initial slope line indicating vesicle formation. The intersection of these two lines determines the CVC.

### Estimation of activation energy to DA vesicle growth process

To estimate the activation energy of the DA molecule for incorporation into the vesicle membrane based on Eq. (5), we measured the average vesicle growth rate, ${v}_{D}$, as a function of temperature. We added DA solution to the DA SUV suspension for two hours and the vesicle growth rate was obtained by measuring mean vesicle size before and after the addition using the dynamic light scattering (DLS) technique.

For each measurement, the parent DA SUV suspension was divided into two portions. One portion was used to measure the initial vesicle size at a set temperature (20, 35, 40, or 45 °C). The other portion was subjected to a continuous co-injection of 100 mM DA solution (4.8 μL/min) and 100 mM HCl (0.8 μL/min) for two hours at a predetermined temperature, under stirring at 600 rpm using a magnetic stirrer with a temperature-controlled heating plate (DP-1S, AS ONE, Japan). Immediately after the supply was completed, DLS measurement of the sample was performed at the predetermined temperature to determine vesicle size after the addition.

Similar experiments were performed for 100 mM DA solution containing 20 mM GlyGly to determine the temperature dependence of vesicle growth rate in the presence of GlyGly. Note that the measurement at 45 °C was not performed to avoid the effects of peptide denaturation.

## Supplementary information

Transparent Peer Review file

Supplementary Information

Description of Additional Supplementary Files

Supplementary Movie

## Data Availability

The datasets generated during and/or analyzed during the current study are available in the Tohoku University Research Data Lake IZUMI repository, https://nc.rdx.tohoku.ac.jp/s/BMpajXZezJn2Ffe.
