# Supplementary material for: Growth of fatty acid vesicles coupled with amino acid sequences of peptides toward evolvable protocells
Source: Commun Chem. 2026 Apr 30;9:234. doi: 10.1038/s42004-026-02043-1 (PMC13338293; doi:10.1038/s42004-026-02043-1)
Supplement: Supplementary file 2 — Supplementary Information [file 42004_2026_2043_MOESM2_ESM.pdf]

Supplementary Information

Growth of Fatty Acid Vesicles Coupled with Amino Acid Sequences of Peptides toward Evolvable Protocells

Akiko Baba[1], Kazuki Yokoyama[1], Keidai Sato[1], Shuna Asanuma[1], Tomoko Kawahata[1], Ulf Olsson[2], Daisuke Unabara[3], Tasuku Hamaguchi[3], Koji Yonekura[3,4], and Masayuki Imai*[1]

[1] Department of Physics, Graduate School of Science, Tohoku University, Sendai, Japan
[2] Division of Physical Chemistry, Department of Chemistry, Lund University, Lund, Sweden
[3]Institute of Multidisciplinary Research for Advanced Materials, Tohoku University, Sendai 980-8577, Japan
[4]Biostructural Mechanism Laboratory, RIKEN SPring-8 Center, Hyogo 679-5148, Japan
* corresponding author: e-mail imai@bio.phys.tohoku.ac.jp

Contents

Supplementary Figures

Supplementary Figure 1. Direct time-resolved observation of DA vesicle growth under constant feeding.
Supplementary Figure 2. Integrity of DA vesicle membrane examined by leak test.
Supplementary Figure 3. Effects of amino acids on growth of DA vesicles.
Supplementary Figure 4. Effects of dipeptides on growth of DA vesicles.
Supplementary Figure 5. Effects of tripeptides on growth of DA vesicles.
Supplementary Figure 6. Schematic representation of vesicle growth promoted by peptides.
Supplementary Figure 7. Determination of CVC of DA + amino acid and DA + peptide solutions.

Supplementary Tables

Supplementary Table 1. Physical properties of 16 amino acids used in this study.
Supplementary Table 2. Effect of amino acids on growth rate of DA vesicles (fitness), and related properties of vesicle suspensions.

Supplementary Table 3. Effect of dipeptides on growth rate of DA vesicles (fitness), pH of the injection solutions, and pH of vesicle suspensions.

Supplementary Table 4. Effect of replacement of one amino acid residue in dipeptides on fitness.

Supplementary Table 5. Effect of tripeptides and a tetrapeptide on growth rate of fatty acid vesicles (fitness), pH of the injection solutions, and pH of vesicle suspensions.

Supplementary Table 6. Walsh analysis for Fitness *P* of tripeptide.

Supplementary Table 7. CVC and fitness of DA solution in the absence and presence of amino acids and peptides.

Supplementary Table 8. List of amino acids used in this study and their purity.

Supplementary Table 9. The makers, purities, and storage temperatures of peptides used in this study.

Supplementary Table 10. Dependence of pH of NaD solution containing amino acid or peptide on NaD concentration.

References

Supplementary Figures

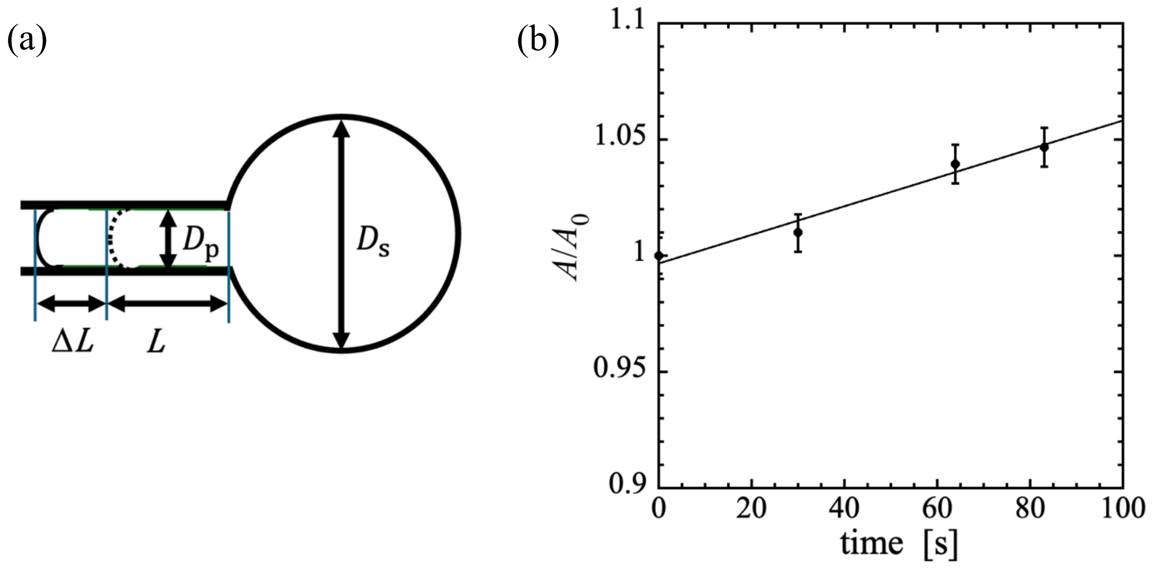

Supplementary Figure 1. Direct time-resolved observation of DA vesicle growth under constant feeding.

To assess vesicle growth dynamics directly, we monitored vesicle growth induced by the ejection of 100 mM DA micellar solution toward a target DA giant vesicle (GV) using optical microscopy (see Direct observation of fatty acid vesicle growth using microinjection technique section in Methods). The vesicle growth process is shown in Supplementary Movie. The geometry of the aspirated vesicle is illustrated in Supplementary Fig. 1a. Changes in vesicle membrane area were calculated from displacements in projection length L inside the pipette using the geometric relations for total area of an aspirated vesicle[1]. Because the pressurized shape of a fluid bilayer vesicle is a perfect sphere, the relations depend only on the diameter D_s of the vesicle-spherical segment outside the pipette, the internal diameter of the pipette D_p (~1 μm), and the projection length L. Thus, total area A is expressed by

$$A = A_{cap} + A_{cyl} + A_{sph},$$

where

$$A_{cap} = \pi D_p^2/2$$

$$A_{cyl} = \pi D_p\left(L - D_p/2\right)$$

$$A_{sph} = \pi D_s^2(1 + u)/2$$

$$u = \left[1 - \left(D_p/D_s\right)^2\right]^{1/2}.$$

The normalized total area A/A_0, where A_0 is the initial vesicle area, is plotted in Supplementary Figure 1b, which shows an approximately linear increase with time. However, DA micellar solutions frequently clogged the ~1 μm pipette tip—particularly when peptides were present—making this approach unsuitable for reproducible long-time injections and thus for quantitative comparisons of growth rates across conditions.

We therefore did not use this approach for quantitative evaluation of vesicle growth. Instead, in the present study we employed a dynamic light scattering (DLS)-based method, which allows statistically robust estimation of vesicle growth.

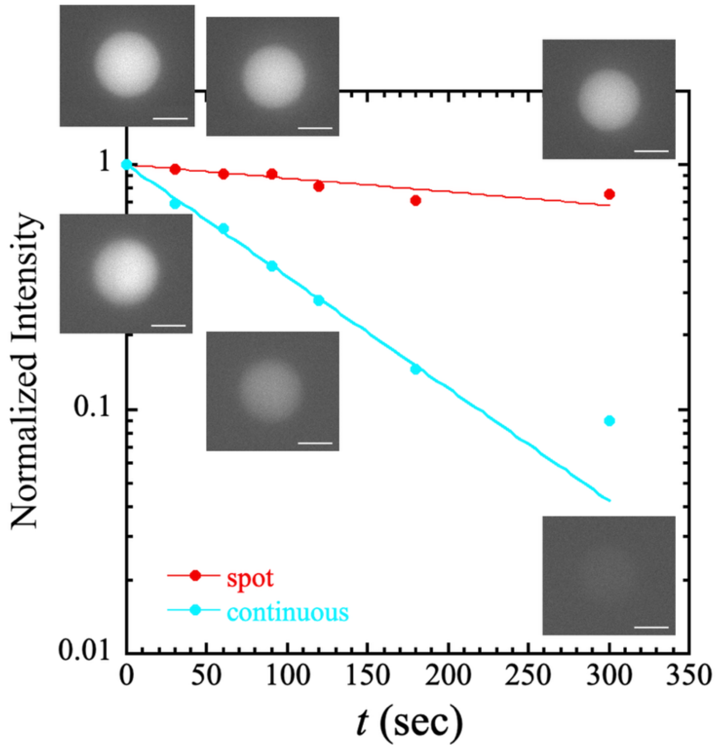

Supplementary Figure 2. Integrity of DA vesicle membrane examined by leak test.

To examine the integrity of the DA vesicle membrane, we performed a leakage assay. The fluorescent dye calcein (λex=495 nm/λem=515 nm, 5 mM) was encapsulated in a DA giant unilamellar vesicle (GUV), and the fluorescence intensity of the encapsulated calcein was monitored over time. When the vesicles were continuously illuminated, the fluorescence intensity decayed exponentially with time (blue circles), and the decay rate was k_{con} = 0.012 s^{-1} obtained by fitting $I(t) = I_0 \exp(-k_{con}t)$. In contrast, when the excitation light was applied for 4 s at discrete time points (0, 30, 60, 90, 120, 180, 300 s) and the sample was kept in the dark between illuminations, the decay rate was k_{spot} = 0.0012 s^{-1}. The ratio k_{spot}/k_{con} ~ 0.1 agrees well with the ratio of irradiation time to total observation time, (4 s × 7)/300 ~ 0.10, indicating that the fluorescence intensity decay was mainly due to photobleaching and that leakage through the membrane was negligible. The inset images show fluorescence micrographs of vesicles at the initial state, after 90 s, and after 300 s. The scale bar is 5 μm. No change in vesicle morphology was observed.

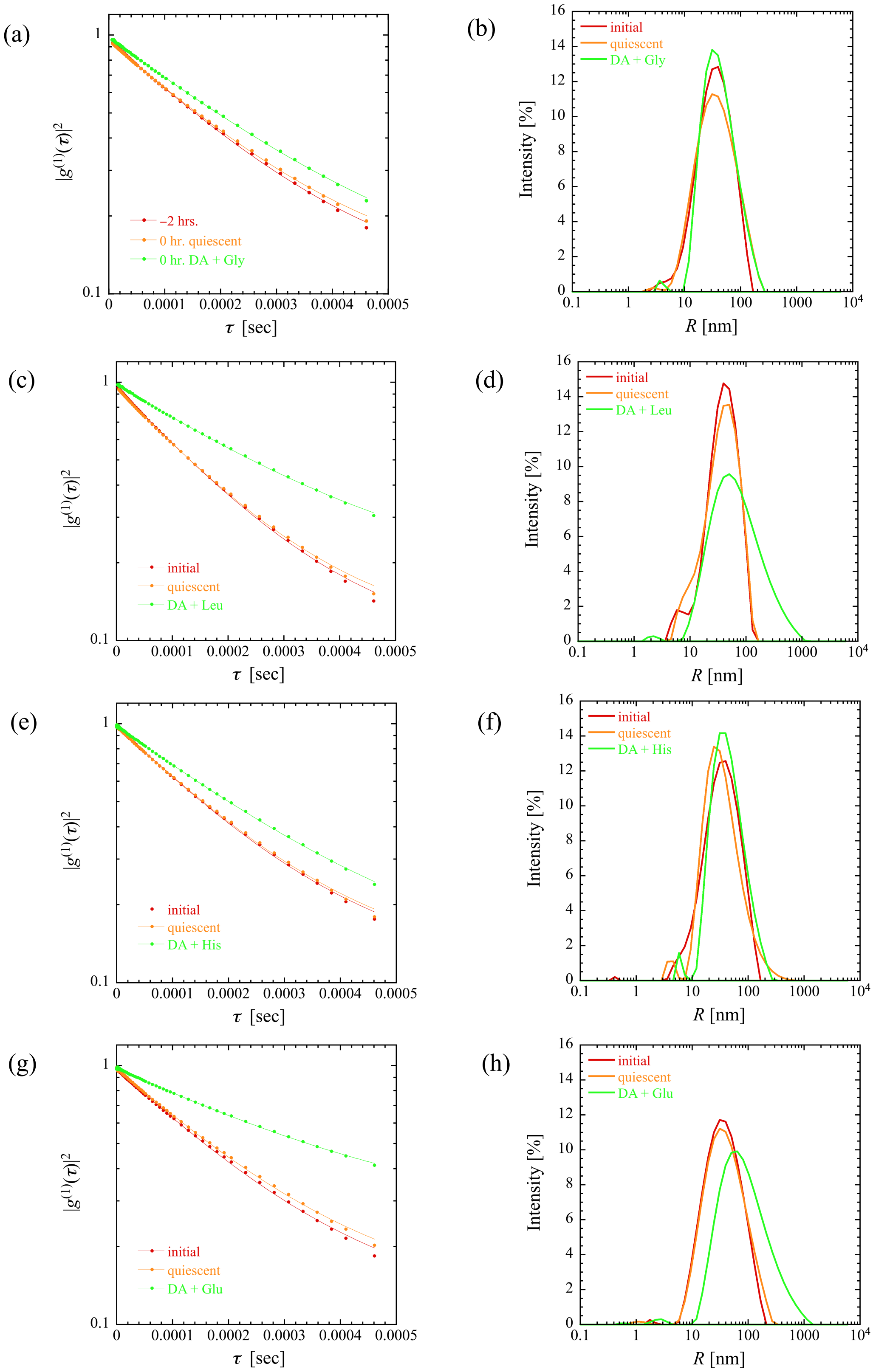
(a)
$|g^{(1)}(\tau)|^2$
τ [sec]
−2 hrs.
0 hr. quiescent
0 hr. DA + Gly
(b)
Intensity [%]
R [nm]
initial
quiescent
DA + Gly
(c)
$|g^{(1)}(\tau)|^2$
τ [sec]
initial
quiescent
DA + Leu
(d)
Intensity [%]
R [nm]
initial
quiescent
DA + Leu
(e)
$|g^{(1)}(\tau)|^2$
τ [sec]
initial
quiescent
DA + His
(f)
Intensity [%]
R [nm]
initial
quiescent
DA + His
(g)
$|g^{(1)}(\tau)|^2$
τ [sec]
initial
quiescent
DA + Glu
(h)
Intensity [%]
R [nm]
initial
quiescent
DA + Glu

Supplementary Figure 3. Effects of amino acids on growth of DA vesicles.

Typical intermediate scattering functions (at q = 2.22 × 10^7 m^{-1}, q: absolute value of the scattering vector) and size distribution functions of DA SUVs at the initial state (t = 0 h), the quiescent state (t = 2 h) and after addition of 100 mM DA and 20 mM amino acid solution (t = 2 h): (a) and (b) for DA and Gly (nonpolar amino acid) solution (DA + Gly), (c) and (d) for DA and Leu (hydrophobic amino acid) solution (DA + Leu), (e) and (f) for DA and His (basic amino acid) solution (DA + His), and (g) and (h) for DA and Glu (acidic amino acid) solution (DA + Glu), respectively. The size distribution functions of (b), (d), (f), and (h) are obtained by inverse Laplace transformation of intermediate scattering functions using a constrained regularization program, CONTIN. Small additional peaks at apparent radii below ~5 nm observed in size distribution functions are not considered to represent vesicles but are attributed to small DA aggregates and/or noise in the DLS analysis.

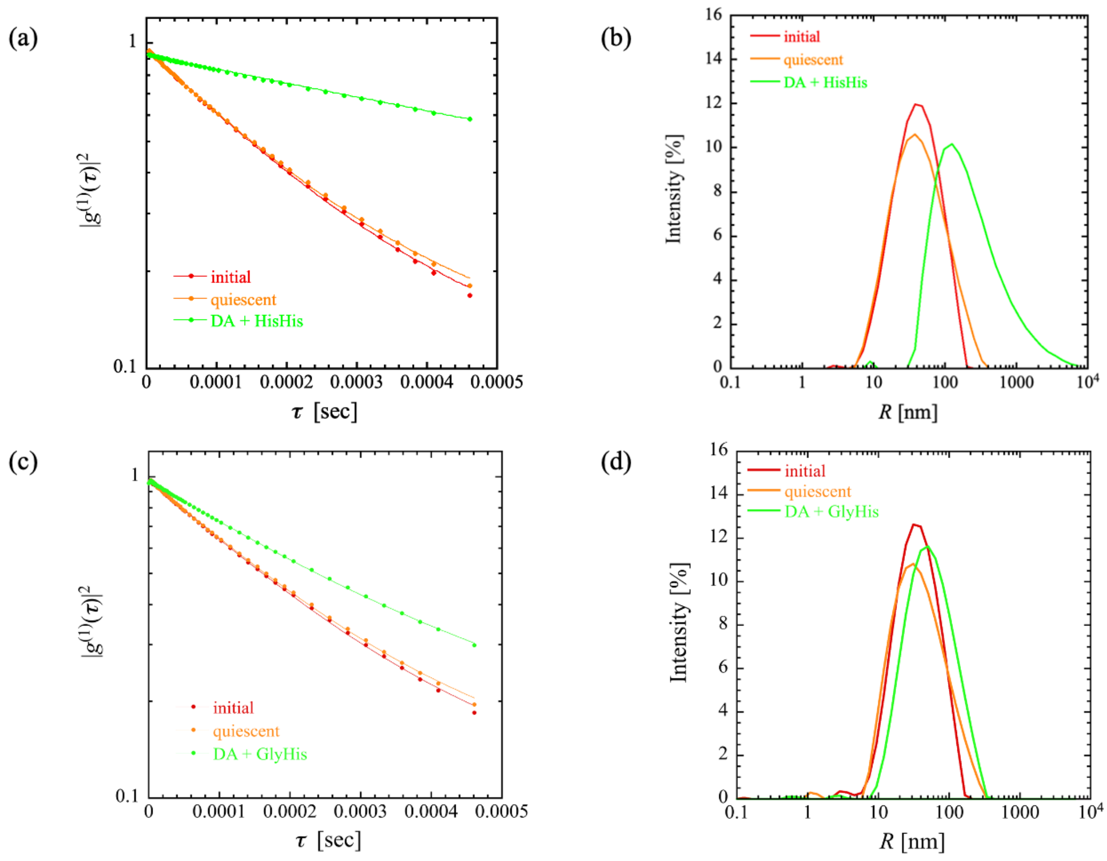

Supplementary Figure 4. Effects of dipeptides on growth of DA vesicles.

Typical intermediate scattering functions (at $q = 2.22 \times 10^7$ m^{-1}) and size distribution functions of DA SUVs at the initial state (t = 0 h), the quiescent state (t = 2 h) and after addition of 100 mM DA and 20 mM dipeptide solution (t = 2 h): (a) and (b) for DA and HisHis solution (DA + HisHis), and (c) and (d) for DA and GlyHis solution (DA + GlyHis), respectively. The size distribution functions of (b) and (d) are obtained by inverse Laplace transformation of intermediate scattering functions using a constrained regularization program, CONTIN. Small additional peaks at apparent size below ~5 nm observed in size distribution functions are not considered to represent vesicles but are attributed to small DA aggregates and/or noise in the DLS analysis.

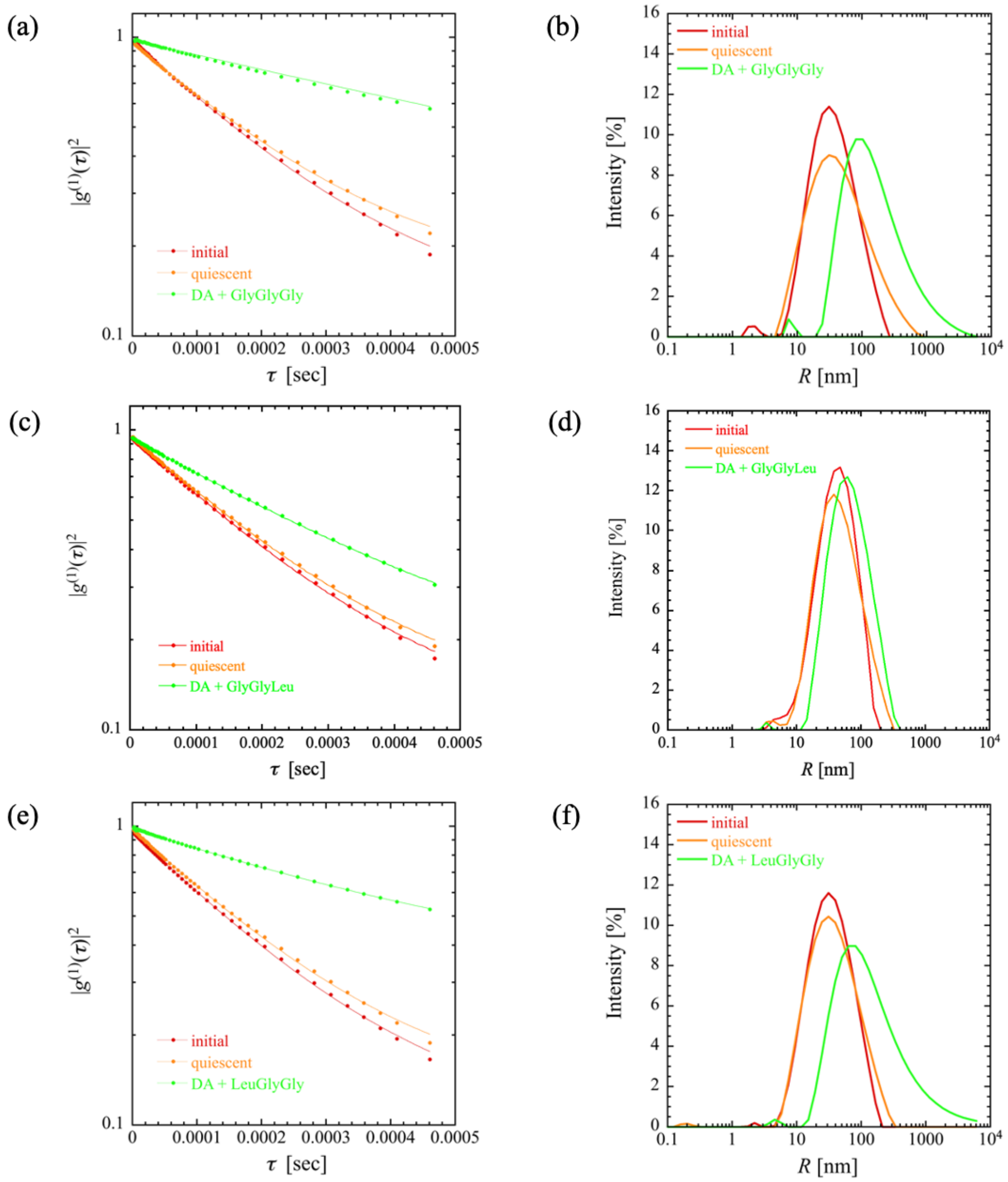

Supplementary Figure 5. Effects of tripeptides on growth of DA vesicles.

Typical intermediate scattering functions (at $q = 2.22 \times 10^7$ m^{-1}) and size distribution functions of DA SUVs at the initial state ($t = 0$ h), the quiescent state ($t = 2$ h) and after addition of 100 mM DA and 20 mM tripeptide solution ($t = 2$ h): (a) and (b) for DA and GlyGlyGly solution (DA + GlyGlyGly), (c) and (d) for DA and GlyGlyLeu solution (DA + GlyGlyLeu), and (e) and (f) for DA and LeuGlyGly solution (DA + LeuGlyGly), respectively. The size distribution functions of (b), (d), and (f) are obtained by inverse Laplace transformation of intermediate scattering functions using a constrained regularization program, CONTIN. Small additional peaks at

apparent size below ~5 nm observed in size distribution functions are not considered to represent vesicles but are attributed to small DA aggregates and/or noise in the DLS analysis.

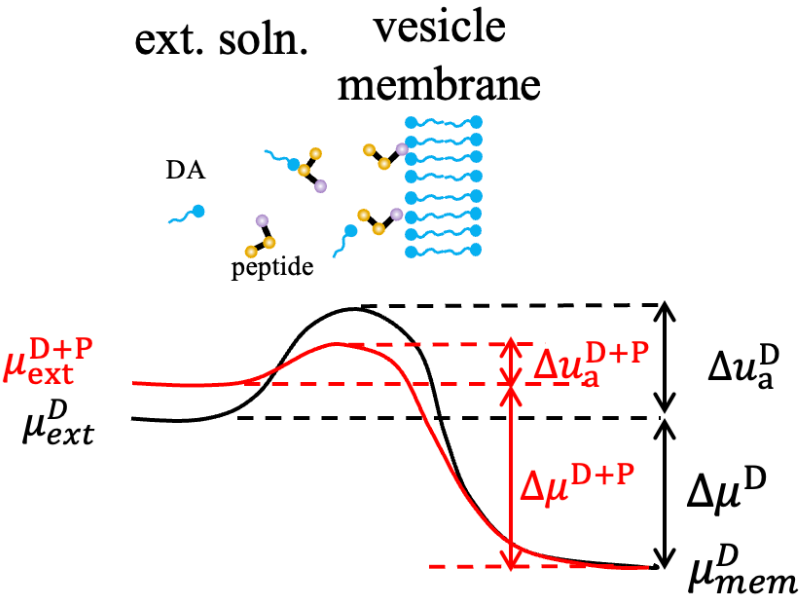

Supplementary Figure 6. Schematic representation of vesicle growth promoted by peptides.

Schematic free energy landscape against a DA molecule incorporation coordinate. Black and red profiles represent the incorporation process in the absence and presence of peptides, respectively. For the black profile, the left side shows the chemical potential of a DA molecule in the external solution, $\mu_{\mathrm{ext}}^{\mathrm{D}}$, and the right side shows the chemical potential of a DA molecule in the vesicle membrane (approximately half of the carboxylic groups are protonated[2]), $\mu_{\mathrm{mem}}^{\mathrm{D}}$. Both states are connected via an activated state with the activation energy per molecule of $\Delta u_{\mathrm{a}}^{\mathrm{D}}$. To attain vesicle growth by incorporating DA molecules in the external solution into the DA vesicle membrane, it is necessary that 1) the chemical potential of DA molecule in the external solution, $\mu_{\mathrm{ext}}^{\mathrm{D}}$, is larger than that in the vesicle membrane, $\mu_{\mathrm{mem}}^{\mathrm{D}}$, *i.e.*, $\Delta\mu^{\mathrm{D}} = \mu_{\mathrm{ext}}^{\mathrm{D}} - \mu_{\mathrm{mem}}^{\mathrm{D}} > 0$, which causes the flux of DA molecules from the external solution to the membrane, and 2) the energy barrier for DA molecules to be incorporated into the membrane, $\Delta u_{\mathrm{a}}^{\mathrm{D}}$, can be overcome with the thermal energy. When the vesicle growth is promoted by peptides, the binding of the peptide to the DA molecule modifies the chemical potential, $\mu_{\mathrm{ext}}^{\mathrm{D+P}}$, and the activation energy $\Delta u_{\mathrm{a}}^{\mathrm{D+P}}$, as shown by the red profile.

For a DA molecule to incorporate from external solution to membrane, the rate of incorporation is proportional to the probability to reach activated state. The fraction of DA molecules in external solution state and in activated state is expressed by

$$\frac{n_{\mathrm{ext}}^{\mathrm{act}}}{N_{\mathrm{ext}}} = e^{-\Delta u_{\mathrm{a}}^{\mathrm{D}}/k_{\mathrm{B}}T}, \qquad \text{(S1)}$$

where N_{ext} and $n_{\mathrm{ext}}^{\mathrm{act}}$ are the number of DA molecules in external solution and in activated state, respectively. Similarly, the fraction of DA molecules in membrane and in activated state is expressed by

$$\frac{n_{\mathrm{mem}}^{\mathrm{act}}}{N_{\mathrm{mem}}} = e^{-(\Delta u_{\mathrm{a}}^{\mathrm{D}}+\Delta\mu^{\mathrm{D}})/k_{\mathrm{B}}T}, \tag{S2}$$

where N_{mem} and $n_{\mathrm{mem}}^{\mathrm{act}}$ are the number of DA molecules in membrane state and in activated state, respectively. Then, the vesicle growth rate is given by

$$\mathrm{Rate} \propto \frac{n_{\mathrm{ext}}^{\mathrm{act}}}{N_{\mathrm{ext}}} - \frac{n_{\mathrm{mem}}^{\mathrm{act}}}{N_{\mathrm{mem}}} = e^{-\Delta u_{\mathrm{a}}^{\mathrm{D}}/k_{\mathrm{B}}T}\left(1 - e^{-\Delta\mu^{\mathrm{D}}/k_{\mathrm{B}}T}\right). \tag{S3}$$

By introducing the constant ν proportional to the attempt frequency to overcome the energy barrier per second, the normalized growth rate is expressed by

$$v_{\mathrm{D}} = \nu e^{-\Delta u_{\mathrm{a}}^{\mathrm{D}}/k_{\mathrm{B}}T}\left(1 - e^{-\Delta\mu^{\mathrm{D}}/k_{\mathrm{B}}T}\right), \tag{S4}$$

which is Eq. (4) in the main text.

As shown in Fig. 4a in the main text, the temperature dependence of the normalized growth rate of DA vesicles gives $\Delta u_{\mathrm{a}}^{\mathrm{D}} = 0.3 \pm 0.3\ k_{\mathrm{B}}T$. Thus, the growth rate is probably governed by the difference in chemical potential of the DA molecule in the external solution and in the vesicle membrane, $\Delta\mu^{\mathrm{D}}$, (Fick's law). At equilibrium, the chemical potential of DA unimer in the external aqueous phase is equal to that of DA molecules within the vesicle membrane, *i.e.* $\mu_{\mathrm{ext}}^{\mathrm{D}} = \mu_{\mathrm{mem}}^{\mathrm{D}}$, and no net incorporation of DA molecules into the membrane occurs. Under these conditions, the unimer concentration in the external solution is equal to CVC. When additional DA molecules are supplied into the system, the chemical potential of DA unimer increases, creating a positive chemical potential difference, $\Delta\mu^{\mathrm{D}} > 0$. This drives the excess unimers to incorporate into the vesicle membrane, resulting in vesicle growth.

Importantly, $\mu_{\mathrm{ext}}^{\mathrm{D}}$ is not determined solely by concentration, but also by molecular interactions of the DA molecule with the surrounding molecules, since $\mu_{\mathrm{ext}}^{\mathrm{D}}$ is expressed by $\mu_{\mathrm{ext}}^{D} = \mu_{\mathrm{ext}}^{0(D)} + k_{\mathrm{B}}T\ln(X_{\mathrm{ext}}^{D})$, where $\mu_{\mathrm{ext}}^{0(D)}$ is the standard chemical potential of DA unimer (interaction energy between DA unimer and surrounding molecules) and X_{ext}^{D} is the mole fraction of DA unimers in the external solution. Therefore, vesicle growth can be promoted by modulation of the molecular interaction to increase $\mu_{\mathrm{ext}}^{\mathrm{D}}$. For example, protonation of decanoate to decanoic acid reduces its hydrophilicity, making it less stabilized in water. This increases $\mu_{\mathrm{ext}}^{\mathrm{D}}$, creating $\Delta\mu^{\mathrm{D}} > 0$, and drives the incorporation of decanoic acid into the membrane, resulting in vesicle growth.

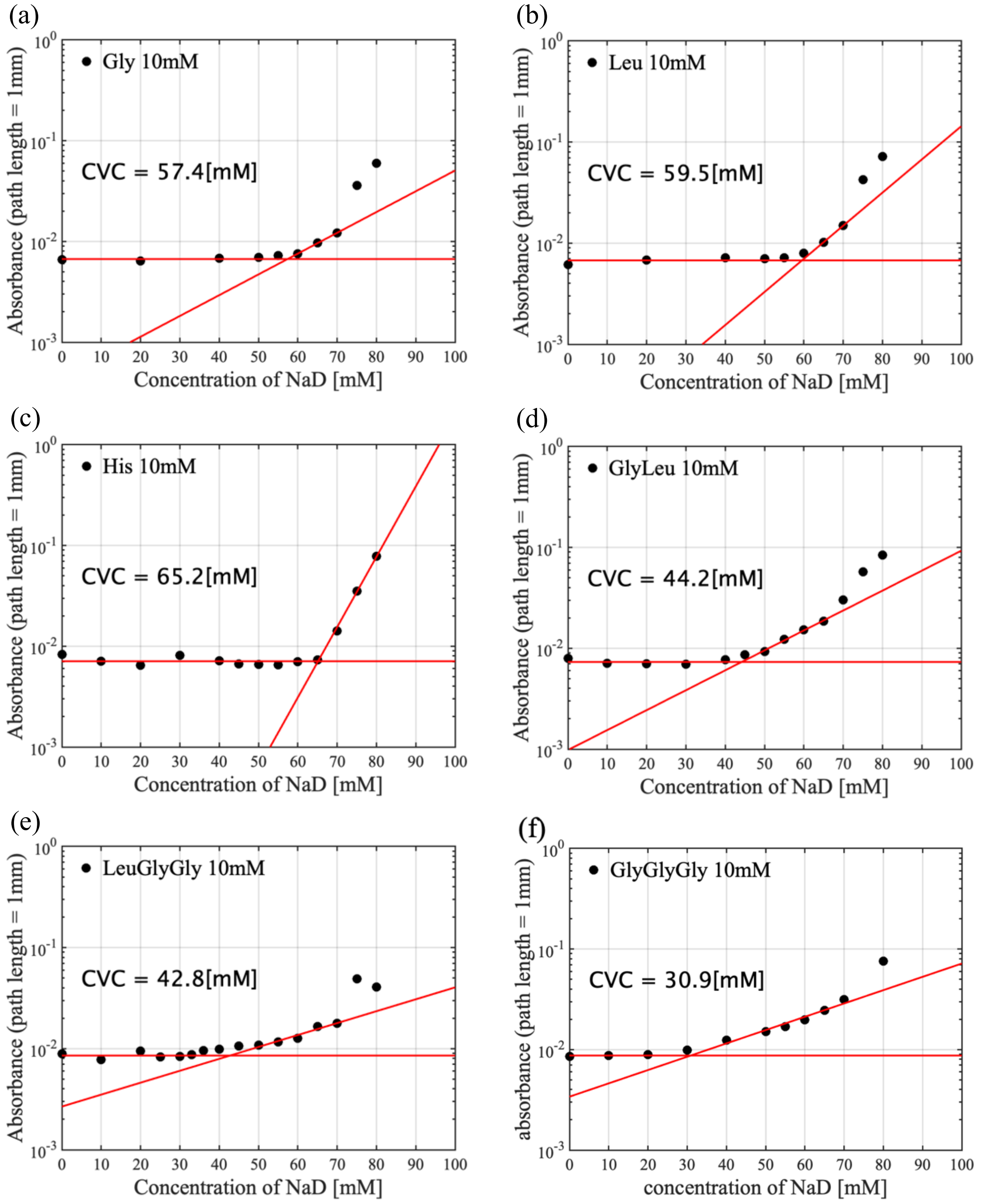

Supplementary Figure 7. Determination of CVC of DA + amino acid and DA + peptide solutions.

Dependence of absorbance (λ = 490 nm) of DA solution containing (a) 10 mM Gly, (b) 10 mM Leu, (c) 10 mM His, (d) 10 mM GlyLeu, (e) 10 mM LeuGlyGly, and (f) 10 mM GlyGlyGly on concentration of sodium decanoate (NaD) at 20 °C. The intersection of flat baseline and initial slope line determines CVC of DA solution in the presence of amino acids or peptides.

Supplementary Tables

Supplementary Table 1. Physical properties of 16 amino acids used in this study. Hydrophobicity[3], dissociation constants for carboxyl group (pK_1) and amino group (pK_2)[4], isoelectric point (pI)[5], and molecular weight (MW) are listed. The correlation coefficients (C.C.) between fitness *P* and physical properties are summarized in the bottom row.

Amino Acid	Hydrophobicity	pK_1	pK_2	pI	MW
Lys	–3.9	2.18	8.95	9.74	146.19
His	–3.2	1.82	8.95	7.59	155.16
Arg	–4.5	2.17	9.04	10.76	174.2
Ser	–0.8	2.21	9.15	5.68	105.09
Thr	–0.7	2.09	9.10	5.60	119.12
Asn	–3.5	2.02	8.80	5.41	132.1
Gln	–3.5	2.17	9.13	5.65	146.15
Asp	–3.5	1.88	9.60	2.77	133.1
Glu	–3.5	2.19	9.67	3.22	147.13
Pro	–1.6	1.99	10.6	6.30	115.1
Trp	–0.9	2.83	9.39	5.89	204.23
Gly	–0.4	2.34	9.60	5.97	75.05
Ala	1.8	2.34	9.69	6.00	89.1
Leu	3.8	2.36	9.60	5.98	131.18
Val	4.2	2.32	9.62	5.96	117.15
lle	4.5	2.36	9.60	6.02	131.18
C.C.	0.15	–0.16	0.36	–0.77	–0.19

Supplementary Table 2. Effect of amino acids on growth rate of DA vesicles (fitness), and related properties of vesicle suspensions.

The first column indicates the type of amino acids and Ctrl means no amino acid. Dimensionless fitness $P = 7200p$ estimated from Eq. (2), standard errors of the mean (SEM) of P, pH of injection solution (100 mM DA micellar solution containing 20 mM amino acid), pH of DA vesicle suspension just after injection of DA micellar solution containing amino acid, pH of mother DA vesicle suspension without injection, ζ-potential of DA vesicles after injection, SEM of ζ-potential, and number of ζ-potential experiments (No. of expt.) are listed for examined 16 amino acids. SEM for the dimensionless fitness P were estimated from three different experiments except for Ctrl and Ile, which were estimated from five and four independent measurements, respectively. SEM for ζ-potentials were estimated from more than three independent experiments shown in No. of expt. column.

Amino Acid	P	SEM	pH of injection	pH with supply T = 0	pH without supply T = 0	ζ- potential [mV]	SEM [mV]	No. of expt.
Ctrl	0.000	0.092	9.9	7.6	7.7	–62.3	0.57	3
Lys	–0.428	0.040	10.1	7.6	7.9			
His	–0.561	0.009	8.3	7.6	7.7	–67.9	2.00	3
Arg	–0.797	0.026	10.6	7.6	7.9			
Ser	–0.085	0.035	8.2	7.6	7.6			
Thr	–0.189	0.022	8.2	7.6	7.7			
Asn	–0.331	0.015	8.1	7.6	7.7			
Gln	–0.339	0.084	8.2	7.6	7.6			
Asp	0.959	0.058	7.9	7.7	7.5			
Glu	0.631	0.026	7.7	7.6	7.5	–64.6	0.70	2
Pro	–0.218	0.016	9.0	7.6	7.6	–69.0	2.44	3
Trp	–0.433	0.021	8.5	7.7	7.7			
Gly	–0.283	0.044	8.4	7.7	7.7	–63.8	1.51	3
Ala	–0.423	0.038	8.5	7.7	7.6	–62.7	7.02	9
Leu	–0.182	0.181	8.5	7.7	7.7	–64.8	1.10	6
Val	0.484	0.129	8.4	7.6	7.7	–65.6	4.67	4
Ile	–0.012	0.007	8.5	7.6	7.7	–61.8	4.27	4

Supplementary Table 3. Effect of dipeptides on growth rate of DA vesicles (fitness), pH of the injection solutions, and pH of vesicle suspensions.

The first column indicates the type of dipeptides, where LeuGly represents the sequence H_2N-Leu-Gly-COOH, and the second column represents dipeptides using the index Leu = 0, Gly = 1, Glu = 2, and His = 3. Dimensionless fitness P, SEM of P, pH of the injection solution (100 mM DA + 20mM dipeptide solution), pH of DA vesicle suspension just after injection of DA + dipeptide solution, and pH of mother DA vesicle suspension without the injection, are listed for examined 11 dipeptides. SEM for the fitness P were estimated from three different experiments. The correlation coefficients (C.C.) between fitness P and pH of the injection solution, pH of DA vesicle suspension just after injection of DA micellar solution containing dipeptide, pH of mother DA vesicle suspension without the injection, are summarized in the bottom row. Note that in the fitness landscape analysis we assumed the commutative property $P_{\mathrm{AB}} = P_{\mathrm{BA}},\ A, B \in \{\mathrm{Leu}, \mathrm{Gly}, \mathrm{Glu}, \mathrm{His}\}$.

Dipeptide	Index	Fitness P	SEM	pH of injection	pH with supply $t = 0$	pH without supply $t = 0$
LeuLeu	00	–0.47	0.18	7.93	7.61	7.61
LeuGly	10	0.69	0.29	7.98	7.67	7.66
LeuGlu	20	0.47	0.23	7.63	7.59	7.68
LeuHis	30					
GlyLeu	01	0.38	0.08	7.96	7.65	7.65
GlyGly	11	5.90	0.44	7.94	7.66	7.66
GlyGlu	21	0.21	0.06	7.70	7.46	7.58
GlyHis	31	–0.05	0.26	8.06	7.64	7.61
GluLeu	02					
GluGly	12					
GluGlu	22	0.47	0.19	7.18	7.30	7.63
GluHis	32	3.17	0.53	7.33	7.28	7.61
HisLeu	03	–0.37	0.08	7.91	7.58	7.62
HisGly	13					
HisGlu	23					
HisHis	33	7.74	0.63	7.49	7.39	7.65
C.C.				–0.28	–0.28	0.33

Supplementary Table 4. Effect of replacement of amino acid residue in dipeptides on fitness

Effect of replacement of one amino acid residue in dipeptides composed of Leu, Gly, Glu and His on the fitness are summarized in Tables (a), (b), (c) and (d), respectively, where amino acid residues in the dipeptide are represented using the index Leu = 0, Gly = 1, Glu = 2, and His = 3. Since the effect of replacement of amino acid residue on the fitness depends not only on the type of amino acid residue being replaced but also on the type of adjacent amino acid residue, the contribution of each amino acid residue to the fitness of dipeptide is obtained by averaging all possible sequences for the remaining positions in the dipeptides (schema average fitness). For simplicity, we assumed that peptide fitness is symmetric with respect to residue order, *i.e.*, $P_{AB} = P_{BA}$, where $A, B \in \{\mathrm{Leu, Gly, Glu, His}\}$. Based on this assumption, we assigned the following fitness values: P_{10} was used in place of P_{01}, P_{02} for P_{20}, P_{30} for P_{03}, P_{12} for P_{21}, P_{13} for P_{31}, and P_{23} for P_{32}. The effect of replacement of amino acid residue on fitness is calculated as follows. For example, in Table (a), replacement to Leu (0), the first row calculates the change in the fitness when the adjacent amino acid residue is Leu (0), *i.e.*, the average of $\Delta P_{10\to 00} = -0.86$, $\Delta P_{20\to 00} = -0.94$, and $\Delta P_{30\to 00} = -0.10$, which is expressed by $\Delta P_{X0\to 00} = -0.63$. Here X represents one of the three amino acid residues other than the amino acid residue to be replaced. The second, third and fourth rows calculate the changes in the fitness when the adjacent amino acid residues are Gly (1), Glu (2), and His (3), respectively. The last column, $\Delta P(\mathrm{Leu}) = -1.77$, is the average of the four row values of $\Delta P_{X*\to 0*}$, where * represents any of four amino acid residues.

Effect of amino acid residue next to the replaced amino acid residue on the fitness is estimated as follows. In the case of Table (a), the column $\Delta P_{1*\to 0*}$ represents effect of the adjacent amino acid residue on the replacement from Gly to Leu. If adjacent amino acid residues do not affect the fitness, each row gives the same value. The highly dispersed ΔP values observed in each column indicate that the effect of replacing one amino acid residue in a dipeptide on fitness depends on the type of adjacent amino acid residue, *i.e.*, epistasis.

(a)

*	$\Delta P_{1*\to 0*}$	$\Delta P_{2*\to 0*}$	$\Delta P_{3*\to 0*}$	$\Delta P_{X*\to 0*}$	$\Delta P(\text{Leu})$
0	$P_{00} - P_{10} = -0.86$	$P_{00} - P_{20} = -0.94$	$P_{00} - P_{30} = -0.10$	–0.63	–1.77
1	$P_{01} - P_{11} = -5.52$	$P_{01} - P_{21} = 0.17$	$P_{01} - P_{31} = 0.43$	–1.64	
2	$P_{02} - P_{12} = 0.23$	$P_{02} - P_{22} = 0.00$	$P_{02} - P_{32} = -2.70$	–0.81	
3	$P_{03} - P_{13} = -0.33$	$P_{03} - P_{23} = -3.54$	$P_{03} - P_{33} = -8.12$	–4.00	

(b)

*	$\Delta P_{0*\to 1*}$	$\Delta P_{2*\to 1*}$	$\Delta P_{3*\to 1*}$	$\Delta P_{X*\to 1*}$	$\Delta P(\text{Gly})$
0	$P_{10} - P_{00} = 0.86$	$P_{10} - P_{20} = -0.09$	$P_{10} - P_{30} = 0.76$	0.51	0.38
1	$P_{11} - P_{01} = 5.52$	$P_{11} - P_{21} = 5.69$	$P_{11} - P_{31} = 5.95$	5.72	
2	$P_{12} - P_{02} = -0.25$	$P_{12} - P_{22} = -0.25$	$P_{12} - P_{32} = -2.95$	–1.15	
3	$P_{13} - P_{03} = 0.33$	$P_{13} - P_{23} = -3.22$	$P_{13} - P_{33} = -7.79$	–3.56	

(c)

*	$\Delta P_{0*\to 2*}$	$\Delta P_{1*\to 2*}$	$\Delta P_{3*\to 2*}$	$\Delta P_{X*\to 2*}$	$\Delta P(\text{Glu})$
0	$P_{20} - P_{00} = 0.94$	$P_{20} - P_{10} = 0.09$	$P_{20} - P_{30} = 0.84$	0.62	–0.47
1	$P_{21} - P_{01} = -0.17$	$P_{21} - P_{11} = -5.69$	$P_{21} - P_{31} = 0.26$	–1.87	
2	$P_{22} - P_{02} = 0.00$	$P_{22} - P_{12} = 0.25$	$P_{22} - P_{32} = -2.70$	–0.82	
3	$P_{23} - P_{03} = 3.54$	$P_{23} - P_{13} = 3.21$	$P_{23} - P_{33} = -4.58$	0.73	

(d)

*	$\Delta P_{0*\to 3*}$	$\Delta P_{1*\to 3*}$	$\Delta P_{2*\to 3*}$	$\Delta P_{X*\to 3*}$	$\Delta P(\text{His})$
0	$P_{30} - P_{00} = 0.10$	$P_{30} - P_{10} = -0.76$	$P_{30} - P_{20} = -0.84$	–0.50	1.73
1	$P_{31} - P_{01} = -0.43$	$P_{31} - P_{11} = -5.95$	$P_{31} - P_{21} = -0.26$	–2.21	
2	$P_{32} - P_{02} = 2.70$	$P_{32} - P_{12} = 2.95$	$P_{32} - P_{22} = 2.70$	2.79	
3	$P_{33} - P_{03} = 8.12$	$P_{33} - P_{13} = 7.79$	$P_{33} - P_{23} = 4.58$	6.83	

Supplementary Table 5. Effect of tripeptides and a tetrapeptide on growth rate of DA vesicles (fitness), pH of the injection solutions and pH of vesicle suspensions.

The first column indicates the type of tripeptides and a tetrapeptide, and the second column represents peptides using the index Leu = 0, Gly = 1, and His = 3. Dimensionless fitness *P*, SEM of *P*, number of experiments for DLS measurements to estimate the fitness *P* (No. of expt.), pH of the injection solution (100 mM DA + 20mM tripeptide or tetrapeptide solution), pH of DA vesicle suspension just after injection of DA + peptide solution, and pH of mother DA vesicle suspension without the injection, are listed for examined 9 tripeptides and 1 tetrapeptide. The SEM of fitness *P* were estimated from 3 - 6 independent experiments shown in No. of expt. column.

Tripeptide	Index	Fitness *P*	SEM	No. of expt.	pH of injection	pH with supply $t = 0$	pH without supply $t = 0$
LeuLeuLeu	000	−0.25	0.11	3	7.99	7.67	7.60
GlyLeuLeu	001	−0.09	0.14	3	8.02	7.66	7.67
LeuGlyLeu	010	0.05	0.08	3	7.98	7.53	7.54
GlyGlyLeu	011	−0.04	0.11	3	8.03	7.67	7.65
LeuLeuGly	100	−0.33	0.09	3	7.96	7.56	7.55
GlyLeuGly	101	0.41	0.17	3	8.03	7.57	7.59
LeuGlyGly	110	4.24	0.95	4	7.98	7.66	7.65
GlyGlyGly	111	5.65	1.03	6	7.92	7.66	7.66
GlyGlyHis	113	7.99	1.06	4	7.95	7.62	7.58
GlyGlyGlyGly	1111	4.41	0.43	5	7.81	7.55	7.57

Supplementary Table 6. Walsh analysis for Fitness *P* of tripeptide.

Dimensionless fitness *P* and Walsh coefficients are listed for examined 8 tripeptides composed of Leu and Gly, *i.e.*, a combinatorially complete set. Tripeptides are represented using the index Leu = L = 0, and Gly = G = 1. First Walsh coefficients are shown in green, second Walsh coefficients in yellow, and third Walsh coefficients in blue. The representation for each Walsh coefficient is given below the table.

Index	Tripeptide	Fitness P	Walsh Coefficient	
000	LLL	−0.25	ε_{***}	+1.20
001	GLL	−0.09	ε_{**1}	+0.56
010	LGL	0.05	ε_{*1*}	+2.54
011	GGL	−0.04	ε_{*11}	+0.21
100	LLG	−0.33	ε_{1**}	+2.57
101	GLG	0.41	ε_{1*1}	+1.05
110	LGG	4.24	ε_{11*}	+4.73
111	GGG	5.65	ε_{111}	+0.91

1st Walsh Coefficient

$e_A = W_A - W_{w.t.}$

ε_{**1} : 000→001 010→011 100→101 110→111

ε_{*1*} : 000→010 001→011 100→110 101→111

ε_{1**} : 000→010 001→011 100→110 101→111

2nd Walsh Coefficient

$$e_{AB} = W_{AB} - [W_{w.t.} + (W_A - W_{w.t.}) + (W_B - W_{w.t.})]$$
$$= W_{w.t.} - W_A - W_B + W_{AB}$$

ε_{*11} : 000→011 100→111

ε_{1*1} : 000→101 010→111

ε_{11*} : 000→110 001→111

3rd Walsh Coefficient

$$e_{ABC} = W_{ABC} - [W_{w.t.} + (W_A - W_{w.t.}) + (W_B - W_{w.t.}) + (W_C - W_{w.t.}) - (W_{AB} - W_{w.t.}) - (W_{AC} - W_{w.t.}) - (W_{BC} - W_{w.t.})]$$
$$= W_{w.t.} - W_A - W_B + W_{AB} - W_C + W_{AC} + W_{AB} - W_{ABC}$$

ε_{111} : 000→111

Supplementary Table 7. CVC and fitness of DA solution in the absence and presence of amino acids and peptides.

CVC, SEM of CVC, number of experiments for CVC measurements (No. of expt.), dimensionless fitness *P*, and SEM of *P* are listed for DA solutions in the absence (Ctrl) and presence of amino acids and peptides. SEM of CVC were estimated from 3 - 5 independent measurements shown in No. of expt. column. Concentrations of amino acids and peptides in CVC measurements were 10 mM.

Sample	CVC [mM]	SEM [mM]	No. of expt.	Fitness *P*	SEM
Ctrl	57.7	0.8	5	0.00	0.05
Gly	58.7	0.7	3	–0.28	0.20
Leu	59.7	0.3	3	–0.18	0.42
His	61.5	1.8	3	–0.56	0.08
GlyLeu	45.7	1.8	3	0.38	0.08
GlyGly	24.5	1.7	3	5.90	0.44
GlyGlyGly	29.3	2.7	4	5.65	1.03
LeuGlyGly	44.5	0.5	3	4.24	0.95

Supplementary Table 8. List of amino acids used in this study and their purity.

Amino Acid	Symbol	Purity
L(+)-Lysine	Lys	95.0 %
L-Histidine	His	98.0 %
L(+)-Arginine	Arg	98.0 %
L-Serine	Ser	99.0 %
L(–)-Threonine	Thr	99.0 %
L(+)-Glutamine	Gln	99.0 %
L-Asparagine Anhydrous	Asn	99 %
L-Glutamic Acid	Glu	99.0 %
L-Aspartic Acid	Asp	99.0 %
Glycine	Gly	99.0 %
L-Alanine	Ala	99.0 %
L-Valine	Val	99.0 %
L-Leucine	Leu	99.0 %
L-Proline	Pro	99.0 %
L(+)-Isoleucine	Ile	99.0 %
L-Tryptophan	Trp	99.0 %

Supplementary Table 9. The suppliers, purities, and storage temperatures of peptides used in this study.

The sequences of peptides are represented the N-terminus on the left and denoted from N to C-terminus. The column “Purity” shows the methods used to determine purity, HPLC stands for high performance liquid chromatography, TA for titrimetric analysis, TLC for thin layer chromatography, and E for elemental analysis**.** The storage temperatures of the peptides are listed in the last column.

Peptide	Sequence	Supplier	Location	Purity	Storage Temperature
GlyGly	Glycyl-glycine	Peptide Institute	Osaka, Japan	≧99.0% HPLC	5℃
GlyLeu	Glycyl-L-leucine	Peptide Institute	Osaka, Japan	≧99.0% HPLC	5℃
GlyHis	Glycyl-L-histidine	Sigma-Aldrich Japan	Tokyo, Japan	≧98% TLC	– 20℃
GlyGlu	Glycyl-L-glutamic acid	Tokyo Chemical Industry	Tokyo, Japan	>98.0% TA	– 20℃
LeuGly	L-Leucyl-glycine	Peptide Institute	Osaka, Japan	≧99.0% HPLC	5℃
LeuLeu	L-Leucyl-L-leucine	Eurofins Genomics	Tokyo, Japan	>98% TLC	– 20℃
LeuGlu	L-Leucyl-L-glutamic acid	Peptide Institute	Osaka, Japan	>99% HPLC	– 20℃
HisLeu	L-Histidyl-L-leucine	Peptide Institute	Osaka, Japan	≧99.0% HPLC	5℃
HisHis	L-Histidyl-L-histidine	Peptide Institute	Osaka, Japan	>98% HPLC	– 20℃
GluHis	L-Glutamyl-L-histidine	Peptide Institute	Osaka, Japan	>99% HPLC	– 20℃
GluGlu	L-Glutamyl-L-glutamic acid	Peptide Institute	Osaka, Japan	≧98.0% HPLC	5℃
GlyGlyGly	Glycyl-glycyl-glycine	Peptide Institute	Osaka, Japan	≧99.0% HPLC	5℃
GlyGlyLeu	Glycyl-glycyl-L-leucine	Peptide Institute	Osaka, Japan	>99% HPLC	– 20℃
GlyLeuGly	Glycyl-L-leucyl-glycine	Peptide Institute	Osaka, Japan	>99% HPLC	– 20℃
LeuGlyGly	L-Leucyl-glycyl-glycine	Peptide Institute	Osaka, Japan	≧99.0% HPLC	5℃
GlyLeuLeu	Glycyl-L-leucyl-L-leucine	Peptide Institute	Osaka, Japan	>99% HPLC	– 20℃
LeuGlyLeu	L-Leucyl-glycyl-L-leucine	Peptide Institute	Osaka, Japan	>99% HPLC	– 20℃
LeuLeuGly	L-Leucyl-L-leucyl-glycine	Peptide Institute	Osaka, Japan	>99% HPLC	– 20℃
LeuLeuLeu	L-Leucyl-L-leucyl-L-leucine	Sigma-Aldrich Japan	Tokyo, Japan	≥90% E	– 20℃
GlyGlyHis	Glycyl-glycyl-L-histidine	Peptide Institute	Osaka, Japan	≧98.0% HPLC	5℃
GlyGlyGlyGly	Glycyl-glycyl-glycyl-glycine	Tokyo Chemical Industry	Tokyo, Japan	>97.0% TA, HPLC	25℃

Supplementary Table 10. Dependence of pH of NaD solution containing amino acid or peptide on NaD concentration.

The pH dependences of the CVC measurement solutions on NaD concentration are tabulated for pure NaD solution (Control) and NaD solution containing 10 mM amino acids (Gly, Leu, and His), and 10 mM peptides (GlyLeu, GlyGly, GlyGlyGly and LeuGlyGly). In the control CVC measurement, the titration volume of HCl to the NaD solution was adjusted so that the pH of the DA solution was approximately 7.8. In the case of CVC measurements in the presence of amino acids or peptides, the same base NaD solution was used as in the control measurement, and the prescribed amount of amino acid or peptide was dissolved in it. As a result, the pH of the NaD solution in the presence of amino acid or peptide varied in the region of low NaD concentration. Notably, no significant pH differences were observed among the peptide-containing samples. It is important to use the same NaD solution for CVC measurements in the absence and presence of amino acids or peptides to ensure that the effect of NaD on the chemical potential of the DA molecule remains the same.

NaD conc. [mM]	Control	Gly	Leu	His	GlyLeu	GlyGly	GlyGlyGly	LeuGlyGly
0	6.8	8.1	7.0	7.8	5.9	6.1	6.0	6.0
10	7.5	-	-	7.6	6.6	6.6	6.6	6.6
20	7.6	8.0	7.6	7.7	6.8	6.8	6.8	6.8
25	7.7	-	-	-	6.9	6.9	6.9	6.9
30	7.7	-	-	7.7	7.0	7.0	7.1	7.1
33	7.7	-	-	-	7.0	7.0	7.1	7.1
36	7.7	-	-	-	7.1	7.1	7.1	7.2
40	7.7	8.0	7.6	7.6	7.2	7.2	7.2	7.3
45	7.7	-	-	7.6	7.2	7.2	7.3	7.3
50	7.8	8.0	7.7	7.6	7.3	7.3	7.3	7.4
55	7.8	7.9	7.7	7.6	7.4	-	7.4	7.5
60	7.7	7.7	7.7	7.6	7.5	7.4	7.5	7.6
65	7.7	7.7	7.7	7.7	7.5	-	7.5	7.6
70	7.7	7.8	7.8	7.8	7.6	7.5	7.6	7.7
75	7.7	7.8	7.8	7.8	7.6	-	7.6	7.7
80	7.7	7.7	7.8	7.7	7.6	7.4	7.5	7.7

Supplementary References

1. Olbrich, K., Rawicz, W., Needham, D., & Evans, E. Water permeability and mechanical strength of polyunsaturated lipid bilayers. *Biophys. J.* **79**, 321-327 (2000).
2. Chen, I. A. & Szostak, J. W. Membrane growth can generate a transmembrane pH gradient in fatty acid vesicles. *Proc. Natl. Acad. Sci. USA* **101**, 7965-7970 (2004).
3. Kyte, J. & Doolittle, R. F. A simple method for displaying the hydropathic character of a protein. *J. Mol. Biol*. **157**, 105-132 (1982).
4. D.R. Lide. *Handbook of Chemistry and Physics*. (CRC Press, 1991).
5. Zimmerman, J. M., Eliezer, N. & Simha, R. The characterization of amino acid sequences in proteins by statistical methods. *J. Theor. Biol*. **21**, 170-201 (1968).
