# Supplementary material for: Growth of fatty acid vesicles coupled with amino acid sequences of peptides toward evolvable protocells
Source: Commun Chem. 2026 Apr 30;9:234. doi: 10.1038/s42004-026-02043-1 (PMC13338293; doi:10.1038/s42004-026-02043-1)
Supplement: Supplementary file 3 — Description of Additional Supplementary Files [file 42004_2026_2043_MOESM3_ESM.pdf]

Description of Additional Supplementary Files:

File name: Supplementary Movie

Description: Movie for direct time-resolved observation of DA vesicle growth under constant feeding (Movie for Fig. 1e).

A DA vesicle was held with a holding pipette, and a 100 mM DA micellar solution was microinjected at a distance of ~20 μm from the vesicle membrane surface. The increase in vesicle membrane area upon micelle feeding was visualized as the displacement of the end of a cylindrical vesicle inside the capillary of the holding pipette. The initial position of the vesicle end (t = 0 s) is indicated by a red arrow and the final position (t = 82 s) is indicated by a green arrow. Experimental details are described in the Direct observation of fatty acid vesicle growth using the microinjection technique section of Methods, and the analysis results are presented in Supplementary Figure 1 (Direct timeresolved observation of DA vesicle growth under constant feeding).
