# Supplementary material for: Growth of fatty acid vesicles coupled with amino acid sequences of peptides toward evolvable protocells
Source: Commun Chem. 2026 Apr 30;9:234. doi: 10.1038/s42004-026-02043-1 (PMC13338293; doi:10.1038/s42004-026-02043-1)
Supplement: Supplementary file 1 — Transparent Peer Review file [file 42004_2026_2043_MOESM1_ESM.pdf]

Reviewer #1 (original Reviewer #4, Remarks to the Author):

The authors have revised the manuscript and made several improvements. Nevertheless, substantial issues remain in the discussion and interpretation of the theoretical model. For these reasons, I cannot recommend publication of the manuscript in its present form.

We deeply appreciate your critical and valuable comments. We have now revised our manuscript paying particular attention to your points and the changes in the text were marked using red font with a flag "**R1-X**, X: comment number of Reviewer #1". In the following we summarized our responses to your comments.

1) *Major concerns regarding Fig. 4a:*

- The axis showing the ratio of growth rates spans nearly two orders of magnitude, whereas the experimental data vary only by a factor of approximately 2–3. Despite this limited variation, it is evident that the DA data do not fall on a straight line, even within the error bars.

We thank the referee for this careful observation. First, we agree that the current axis range is very wide compared to the actual variation of the data. In the revised version, we have adjusted the y-axis limits to focus on the measured range to better visualize the scatter (Fig. R1-1: New Fig. 4a). Here, we plot $\ln(v_{\mathrm{D}}/v_{D}^{0})$ and $\ln(v_{\mathrm{D+GG}}/v_{\mathrm{D+GG}}^{0})$ against $1/T$ for DA vesicle growth in the absence and presence of the peptide GlyGly, where slopes of the Arrhenius type plot for $\Delta u_a = 1\,k_B T_0$ and $3k_B T_0$ (T_0=298 K) are shown for reference.

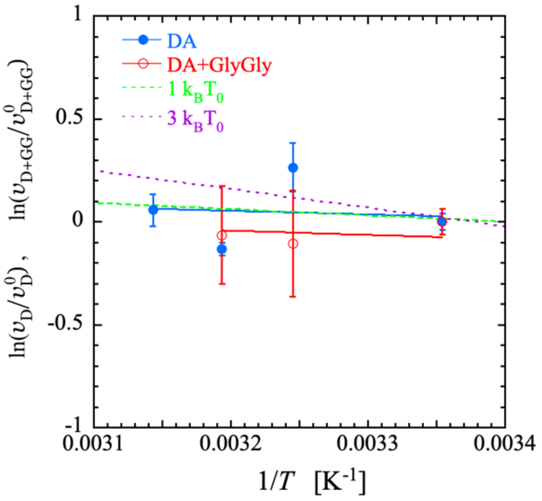

Fig. R1-1 Arrhenius-type analysis of the apparent activation contribution for incorporation of DA molecules into the membrane in the absence and presence of GlyGly. The normalized average growth rates of DA vesicles, expressed as $\ln(v_{\mathrm{D}}/v_{\mathrm{D}}^{0})$ and $\ln(v_{\mathrm{D+GG}}/v_{\mathrm{D+GG}}^{0})$, are plotted against $1/T$ for vesicle growth upon addition of 100 mM DA (blue circles, ctrl) and 100 mM DA + 20 mM GlyGly (red open circles, GlyGly), respectively, where v_{D}^{0} and $v_{\mathrm{D+GG}}^{0}$ are the corresponding normalized growth rates at T_0= 298 K. Error bars indicate SEM from 3–5 independent measurements. Blue and red lines indicate reference trends based on Eqs. (5) and (6), respectively. For comparison, reference lines corresponding to $\Delta u_a = k_B T_0$ and $3k_B T_0$ are also shown.

Second, we agree that the DA data do not exhibit a strong straight-line trend. Importantly, although the experimental data are scattered, the slope is smaller than the straight line of $\Delta u_a = 3k_B T_0$ and on the order of $\Delta u_a = k_B T_0$, which is

consistent with our main conclusion that the activation barrier for DA incorporation is on the order of, or smaller than, the thermal energy, $k_B T$. When the apparent activation energy is close to zero, the expected temperature dependence is weak, and the scatter (including experimental uncertainties) becomes comparatively prominent; consequently, the Arrhenius plot is not expected to show a pronounced linear trend over a limited temperature window.

Finally, we emphasize that the key purpose of Fig. 4a is comparative: the temperature dependences of the normalized vesicle growth rates in the absence and presence of GlyGly are indistinguishable within experimental uncertainty. This supports our conclusion that peptide-enhanced growth is not due to a detectable reduction in the activation barrier, but rather is consistent with an increase in the driving chemical-potential difference.

- The idea of an Arrhenius plot is to extract an exponent from the slope of a well-defined linear relationship. In the presented data, however, no clear slope is visible, and it is therefore not possible to confirm Arrhenius-like behavior.

We thank the referee for this important point.
We agree that the purpose of an Arrhenius plot is to extract an activation energy from a clearly linear relationship over a sufficiently wide dynamic range. In our case, the temperature dependence of the growth rate is weak over the accessible experimental window, which results in a small apparent slope and makes any "Arrhenius-like" linear trend difficult to confirm visually. We have therefore revised the text to avoid overstating Arrhenius behavior and to clarify that our analysis provides, at best, an order-of-magnitude estimate of the activation contribution rather than a precise activation energy. Using this Arrhenius-type analysis, we estimate an apparent value of $\Delta u_{\mathrm{a}}^{D} = 0.3 \pm 0.3\ k_B T_0$, which is statistically consistent with zero and implies $\Delta u_{\mathrm{a}}^{D} \lesssim 1\ k_B T_0$ within our experimental resolution.

Importantly, our primary objective is comparative: applying the same analysis to vesicles in the presence of a representative growth-promoting peptide (GlyGly) yields $\Delta u_{\mathrm{a}}^{\mathrm{D+GG}}$ indistinguishable from $\Delta u_{\mathrm{a}}^{D}$ within uncertainty. Thus, regardless of the limited linearity, the data support our key conclusion that peptide-enhanced growth is not driven by a reduction in the activation barrier, but rather by an increased chemical-potential driving force.

- It is unclear why the quantity v_D/v_D^0 T is plotted instead of v_D/v_D^0. Based on the discussion

around Eq. (5), this equation can be rewritten as v_D = v exp[- Delta u_a^D] ln(X_ext^D/X_cvc^D). To extract u_a^D, it would therefore be more appropriate to present v_D/v_D^0.

We thank the reviewer for pointing this out. We agree that, in the small-$\Delta\mu^{\mathrm{D}}$ regime where $\Delta\mu^{\mathrm{D}} \approx k_B T \ln(X_{\mathrm{ext}}^D / X_{\mathrm{CVC}}^D)$, Eq. (5) can be written as $v_{\mathrm{D}} \approx \nu e^{-\Delta u_{\mathrm{a}}^D / k_{\mathrm{B}} T} \ln(X_{\mathrm{ext}}^D / X_{\mathrm{CVC}}^D)$. In this form, the temperature dependence relevant to the activation term is more appropriately assessed using v_{D}/v_D^0, rather than $(v_{\mathrm{D}}/v_D^0)T$. We have therefore revised Fig. 4a and the corresponding text to plot $\ln(v_{\mathrm{D}}/v_D^0)$ versus $1/T$, and we removed the extra factor *T* (see Fig. R1-1). This change does not affect our conclusion: the apparent slope remains small, indicating that the activation contribution is negligible within experimental resolution, and the peptide-containing case yields an indistinguishable apparent activation contribution within uncertainty.

- Furthermore, if Fig.4 an indeed shows v_D/v_D^0 T this quantity has units of temperature. These units are not indicated on the axis. In addition, there is an inconsistency between the text and the figure: the text states that ln(v_D/v_D^0 T) is plotted, whereas the axis label suggests that v_D/v_D^0 T is shown.

We agree. In the revised version, Fig. 4a now plots $ln(v_{\mathrm{D}}/v_D^0)$ versus $1/T$, rather than $(v_{\mathrm{D}}/v_D^0)T$. As a result, the plotted quantity is dimensionless and the figure, axis label, and text are now fully consistent.

These referee's concerns are addressed in the revised manuscript, *ll*. 414-434 (R1-1), and in the revised and its caption.

2) *Discussion of the flux:*
While it is correct that a flux is driven by a gradient in chemical potential, no clear justification is provided for the assumption that 'transport is localized within an interfacial layer of molecular thickness l'. Moreover, the expression for the flux J is not subsequently used to calculate any quantities or to support any conclusions. I therefore recommend removing the discussion surrounding J, as it currently does not contribute meaningfully to the analysis.

We thank the reviewer for this helpful suggestion. We agree that the current discussion introducing an explicit flux *J* and an interfacial thickness *l* does not directly support any subsequent calculation or conclusion. We have therefore removed the discussion surrounding *J* (including the assumption of a transport layer of thickness *l*) from the revised manuscript.

Importantly, this removal does not affect our main argument. In the revised text, we no longer invoke an explicit spatial transport model. Instead, within the two-state framework, we describe peptide effects phenomenologically through an apparent local chemical potential of DA near the membrane, $\mu_{\mathrm{ext}}^{\mathrm{D+P}}$ (and thus $\Delta\mu^{\mathrm{D+P}}$), which captures how membrane-associated peptides may modify the local driving force for DA uptake, without introducing an explicit spatial coordinate.

The revised discussion is provided in the revised manuscript (*ll.* 487–496: R1-2).

3) Role of membrane-bound peptides:
The authors state: 'Since only a small fraction of the peptides (on the order of a few percent) is adsorbed on the vesicle surface, these membrane-associated peptides are the ones that are directly responsible for modulating DA uptake and hence vesicle growth.'. This argument is not clear to me. If peptides in solution also affect the chemical potential, it is not evident why their contribution to the uptake process can be neglected relative to that of the membrane-bound peptides

We also appreciate the reviewer's point regarding the role of membrane-bound versus soluble peptides. Our main mechanism supported by the experimental data is a bulk effect: peptides in solution lower the CVC and thereby increase the driving chemical-potential difference for DA incorporation, by shifting the external-phase chemical potential of DA from $\mu_{\mathrm{ext}}^{\mathrm{D}}$ to $\mu_{\mathrm{ext}}^{\mathrm{D+}P}$, thereby increasing the driving difference $\Delta\mu^{\mathrm{D+P}} = \mu_{\mathrm{ext}}^{\mathrm{D+P}} - \mu_{\mathrm{mem}}^{\mathrm{D}}$. This contribution is not neglected.
What we intended to convey is that DA incorporation occurs at the membrane interface, so peptides that are membrane-associated are the species most directly positioned to influence the uptake step. However, we agree that our original wording ("directly responsible") was too strong and could be read as implying that soluble peptides are unimportant. We have therefore revised the text to clarify that membrane-bound peptides may contribute at the point of incorporation, while the primary and quantitatively supported effect in this work is the bulk shift in chemical potential (CVC reduction) caused by peptides in solution.

The revised discussion is provided in the revised manuscript (*ll.* 487−496: R1-3).

4) *Finally, it remains unclear which conclusions or insights are genuinely derived from the theoretical model, as opposed to being effectively introduced through assumptions. Clarifying this point is essential in order to assess the explanatory and predictive value of the model.*

We thank the reviewer for raising this important point. We agree that it is essential to distinguish which insights follow from the theoretical framework and which are introduced as assumptions or empirical inputs.

What is derived from the model:

Our two-state growth model describes DA transfer from the external solution to the vesicle membrane as transitions between two effective states, where the growth rate is expressed as

$$v_{\mathrm{D}} = \nu e^{-\Delta u_{\mathrm{a}}^{\mathrm{D}}/k_{\mathrm{B}}T}\left(1 - e^{-\Delta\mu^{\mathrm{D}}/k_{\mathrm{B}}T}\right).$$

This expression separates an activation contribution ($\Delta u_{\mathrm{a}}^{\mathrm{D}}$) from a thermodynamic driving contribution ($\Delta\mu^{\mathrm{D}}$). In the small-$\Delta\mu^{\mathrm{D}}$ regime, the model predicts $v_{\mathrm{D}} \approx \nu e^{-\Delta u_{\mathrm{a}}^{D}/k_{\mathrm{B}}T}(\Delta\mu^{D}/k_{\mathrm{B}}T)$, implying that peptide-induced changes in growth must arise primarily through either (i) a change in the activation barrier or (ii) a change in the driving chemical potential difference.

What is constrained by experiments:

Using the same Arrhenius-type analysis for vesicles with and without peptides, we find no detectable change in the apparent activation contribution within experimental uncertainty. This argues against a barrier-reduction mechanism as the dominant origin of peptide-enhanced growth under our experimental conditions. We therefore measured the peptide-dependent CVC of DA, which serves as an experimental proxy for the chemical potential of DA in the aqueous phase. Across peptide sequences, the fitness correlates strongly with the CVC shift, supporting the conclusion that peptide sequence primarily modulates the thermodynamic driving force for incorporation.

Resulting conclusion and predictive value:

Combining these two constraints within the model leads to the central conclusion that sequence-dependent growth (fitness) differences are governed predominantly by thermodynamic modulation of $\Delta\mu^{\mathrm{D}}$, rather than by kinetic changes in $\Delta u_{\mathrm{a}}^{\mathrm{D}}$. The model therefore yields a testable prediction: peptide sequences that lower the CVC (increase the driving force) should systematically promote faster vesicle growth, while the apparent activation barrier should remain small and largely sequence-insensitive within our experimental regime.

We have revised the manuscript to explicitly delineate these model-derived implications from the experimentally constrained inputs (*ll.* 482-509: R1-4).

We have done our best to rewrite our article according to your suggestions. We hope that with the changes made the achievements obtained in the work are better described so that a broad readership more clearly understands the concepts of the work and the key findings. We appreciate your kind and critical comments very much and hope our paper will be acceptable as revised.

Best regards and kindest wishes,

Masayuki Imai

Department of Physics, Tohoku University

Reviewer #2 (original Reviewer #3, Remarks to the Author):

1) *The authors have added additional experiments to verify the growth of the vesicles. For completeness, the same experiments could be performed with the most "active" peptides, dipeptides and tripeptides. Otherwise, the authors have addressed all of the comments.*

We thank the reviewer for this constructive suggestion. We have now revised our manuscript paying particular attention to your points and the changes in the text were marked using red font with a flag "**R2-1**, 1: comment number of Reviewer #2". In the following we summarized our responses to your comments.

We agree that, ideally, direct single-vesicle injection experiments under strongly growth-promoting peptide conditions would provide an additional demonstration. As a representative example, we attempted this experiment in the presence of one of the most active peptides, GlyGly.

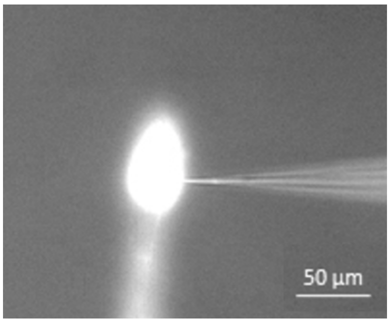

Fig. R2-1. An example of pipette tip clogging due to aggregate formation during microinjection. The flow of the injection solution is visualized using a fluorescent dye.

However, under these conditions, the peptide–fatty-acid interaction markedly lowers the CVC and promotes rapid formation of fatty-acid aggregates near the tip of the pipette (Fig. R2-1), which frequently blocks the ~1 µm outlet and prevents stable, reproducible injection into a held vesicle (distance between the vesicle and the pipette tip: ~20 µm). For this reason, the original micropipette experiment is technically unsuitable for systematic testing under highly active peptide conditions. Therefore, to evaluate peptide effects on vesicle growth quantitatively, we employed dynamic light scattering (DLS), which allows vesicle growth to be compared under nearly homogeneous conditions by gradually adding a small volume of DA micellar solution over two hours while gently stirring the suspension.

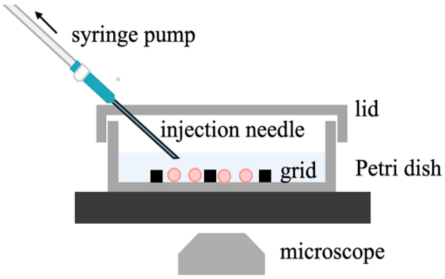

Fig. R2-2. Bulk injection method: DA vesicles were allowed to settle at the bottom of a grid-partitioned dish, and a mixed solution of 100 mM DA micelles and 20 mM GlyGly was supplied from an injection needle at a rate of 2 µL/min.

To nevertheless obtain direct visual confirmation of growth under peptide-containing conditions, we implemented an alternative geometry using an injection needle with an inner diameter of 330 µm and supplied a DA micelle (100 mM) + GlyGly (20 mM)

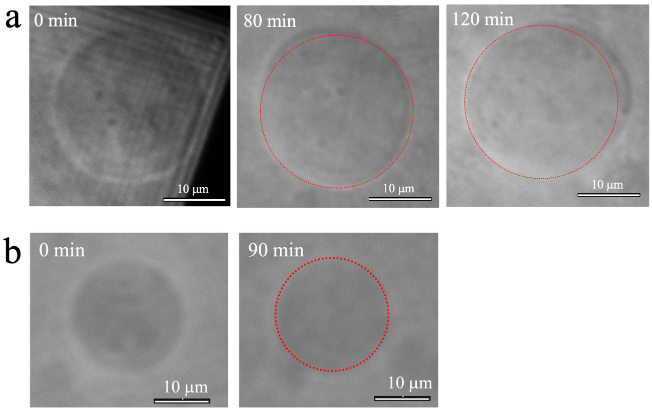

Fig. R2-3. Growth of DA vesicles during bulk injection at 2 μL/min of (a) a 100 mM DA micellar solution and (b) a mixed solution containing 100 mM DA micelles and 20 mM GlyGly. The times shown in each image indicate the elapsed time from the start of injection. Red dashed circles mark the vesicle size at 0 min in each experiment.

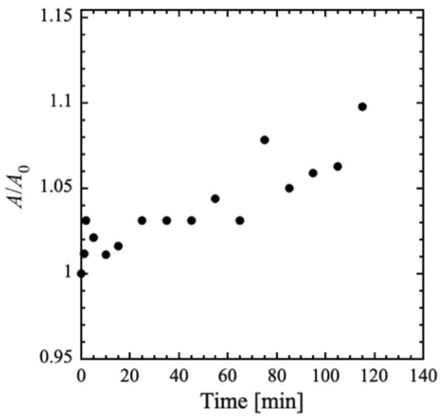

Fig. R2-4. Time evolution of the normalized membrane area of DA vesicles during bulk injection of a mixed solution of 100 mM DA micelles and 20 mM GlyGly at 2 μL/min.

solution from a larger distance (~200 μm) without holding the vesicle (bulk injection setup; Fig. R2-2). In this setup, we observed a measurable membrane-area increase (6–10% over 120 min; Figs. R2-3a and R2-4), confirming that micelle supply can drive vesicle growth also in the presence of GlyGly. The apparent growth rate is much slower than in the original near-field injection experiment, which we attribute to the greatly reduced delivery rate at the larger injection distance. As a control in the same bulk-injection geometry, injecting DA micelles alone produced little or no detectable area increase over the same time window (Fig. R2-3b), consistent with the reduced delivery rate at the larger injection distance. However, quantitatively evaluating vesicle growth rates with this setup would require a thorough exploration of the experimental conditions. Because of these technical limitations and the different delivery geometry, we do not use the injection experiments for quantitative comparison across peptide sequences.

Accordingly, in the present work, the injection experiments are used as direct qualitative demonstrations of micelle-driven membrane growth, whereas peptide-sequence-dependent comparisons are evaluated quantitatively using bulk DLS measurements.

We have also added a note to Supplementary Fig. 1 (R2-1) in the Supplementary Information stating that DA micellar solutions frequently clogged the ~1 μm pipette tip—particularly when peptides were present—making this approach unsuitable for reproducible long-time injections and thus for quantitative comparisons of growth rates across conditions.

We sincerely thank the referees for their careful and constructive feedback. We have addressed all comments and revised the manuscript to improve clarity and strengthen the presentation of our results and conclusions. We hope that the revised manuscript will now be suitable for publication.

Best regards and kindest wishes,

Masayuki Imai

Department of Physics, Tohoku University
